# Mathematical Model of Three Age-Structured Transmission Dynamics of Chikungunya Virus

**DOI:** 10.1155/2016/4320514

**Published:** 2016-04-05

**Authors:** Folashade B. Agusto, Shamise Easley, Kenneth Freeman, Madison Thomas

**Affiliations:** ^1^Department of Ecology and Evolutionary Biology, University of Kansas, Lawrence, KS 66045, USA; ^2^Department of Mathematics and Statistics, Austin Peay State University, Clarksville, TN 37044, USA

## Abstract

We developed a new age-structured deterministic model for the transmission dynamics of chikungunya virus. The model is analyzed to gain insights into the qualitative features of its associated equilibria. Some of the theoretical and epidemiological findings indicate that the stable disease-free equilibrium is globally asymptotically stable when the associated reproduction number is less than unity. Furthermore, the model undergoes, in the presence of disease induced mortality, the phenomenon of backward bifurcation, where the stable disease-free equilibrium of the model coexists with a stable endemic equilibrium when the associated reproduction number is less than unity. Further analysis of the model indicates that the qualitative dynamics of the model are not altered by the inclusion of age structure. This is further emphasized by the sensitivity analysis results, which shows that the dominant parameters of the model are not altered by the inclusion of age structure. However, the numerical simulations show the flaw of the exclusion of age in the transmission dynamics of chikungunya with regard to control implementations. The exclusion of age structure fails to show the age distribution needed for an effective age based control strategy, leading to a one size fits all blanket control for the entire population.

## 1. Introduction

Chikungunya is a viral disease that is transmitted to humans from an infected mosquito of the* Aedes* genus (particularly the* Aedes aegypti* and* Aedes albopictus* mosquitoes [1, 2]). It is an RNA virus that belongs to* Alphavirus* genus of the family Togaviridae [3]. It was first described about 1952 during an outbreak in southern Tanzania [3]. Chikungunya in the Kimakonde language (the language from where the name was derived) means to become contorted or “bend over” [3]. There have been numerous cases of reemergence of chikungunya in Africa, Asia, Europe, and more recently the Caribbean [4]. The virus was isolated in 1960s in Bangkok and in 1964, the virus resurfaced in parts of India including Vellore, Calcutta, and Maharastha [5]. Other outbreaks include Sri Lanka in 1969, Vietnam in 1975, Myanmar in 1975, and Indonesia in 1982 [5]. A large outbreak occurred in the Democratic Republic of the Congo in 1999-2000 [3]. In the years 2005–2007, an outbreak occurred in the islands of the Indian Ocean. Gabon was hit with an outbreak in 2007 [3]. Since 2005, India, Indonesia, Thailand, Maldives, and Myanmar have encountered over 1.9 million cases [3]. The disease spread to Europe by 2007 with 197 cases being recorded [3]. More recently, in December 2013, the French part of the Caribbean island of St. Martin reported two laboratory-confirmed autochthonous (native) cases [3, 4]. Since then, local transmission have been confirmed in the Dutch part of Saint Martin (St Maarten), Anguilla, British Virgin Islands, Dominica, French Guiana, Guadeloupe, Martinique, and St Barthelemy [3]. As of October 2014, over 776,000 suspected cases of chikungunya have been recorded in the Caribbean islands, Latin American countries, and some south American countries [3]. About 152 deaths have also been attributed to the disease during the same period. Mexico and USA have also recorded imported cases. On October 21, 2014, France confirmed four cases of chikungunya locally acquired infection in Montpellier, France [3].

In 2005-2006, a major chikungunya outbreak involving numerous islands in the Indian Ocean (notably La Reunion Island) occurred; one-third of the population were infected [6]. According to Schuffenecker et al. [7] and Vazeille et al. [8], in two concurrent studies, the chikungunya virus strains in the Reunion Island outbreak mutated to facilitate the disease transmission by* Aedes albopictus* (Tiger mosquito) [6, 9]. The mutation was a point mutation in one of the viral envelope genes (E1 glycoprotein gene (E1-226V)) [10, 11]. Dubrulle et al. [6] found that this mutation allowed the virus to be present in the mosquito saliva only two days after the infection, instead of approximately seven days in the* Aedes aegypti* mosquitoes. This shows that* Aedes albopictus* is a slightly more efficient host than* Aedes aegypti* in transmitting the variant E1-226V of chikungunya virus. Hence, this result indicates that other areas where the tiger mosquitoes are present could be at greater risk of outbreak with an enhanced transmission of chikungunya virus by* Aedes albopictus*.

Following an effect bite (i.e., a bite leading to an infection) from infected mosquitoes [1, 2], the incubation period is usually within 3–7 days; symptoms include fever, headache, nausea, fatigue, rash, and severe joint pain (including lower back, ankle, knees, wrists, or phalanges) [1, 2]. There is no antiviral medicine to treat the disease [1, 2]; all the treatments are directed at relieving the disease symptoms [3]. There is no preventative vaccine for chikungunya [3]; however, findings of an experimental vaccine in an early-stage clinical trial are promising; it prompted an immune response in all 25 volunteers [12].

Chikungunya rarely results in death and infected individuals are expected to make full recovery with life-long immunity [2]. However there are some cases where individuals experience joint pains for several months or years after the initial infection [3]. There have also been reports of eye, neurological, and heart complications and gastrointestinal complaints [3]. The disease symptoms, generally, are mild and the infection may go unrecognized; however, several studies [13–16] have shown that children (especially neonates), the elderly (≥65 years), and people with medical conditions (such as high blood pressure, diabetes, or heart disease) have more severe clinical manifestation of chikungunya than in older children and adults (<65 years). The severity of the symptoms can be described by a U-shaped curve, with a maximum occurring in young infants and the elderly and a minimum in older children [16]. Furthermore, the rate of asymptomatic infection among children varies according to different outbreak reports (range 35–40%) [16]; overall, approximately 3–28% of infected individuals will remain asymptomatic [17, 18]. In the study of the 2006-2007 chikungunya epidemic in Kerala, South India, Vijayakumar et al. [19] showed an age distribution of people affected with chikungunya. Their study indicates that the adult group (ages 15–59) were the most affected age group; they consist of about 73.4% of the entire study population; this is followed by the elderly group (ages > 60); this group make up about 15.6% of the study population. Finally, 11% of the cases occurred in persons ages <15 years. Similar age distribution was reported in other epidemics in India [20], Thailand [21], and Reunion Islands [5, 22] and across Europe [23] (including Austria, Czech Republic, Estonia, Finland, France, Germany, Greece, Hungary, Ireland, Italy, Latvia, Lithuania, Luxembourg, Malta, Poland, Romania, Slovakia, Slovenia, Spain, Sweden, and United Kingdom).

A number of studies have been carried out to study the chikungunya virus, considering different factors that effect the outbreak of the disease (see [21, 24–30]). Ruiz-Moreno et al. [24] analyzed the potential risk of chikungunya introduction into the US; their study combines a climate-based mosquito population dynamics stochastic model with an epidemiological model to identify temporal windows that have epidemic risk. Dumont et al. [25] propose a model, including human and mosquito compartments, that is associated with the time course of the first epidemic of chikungunya in Reunion Island. Using entomological results, they investigated the links between the episode of 2005 and the outbreak of 2006. Manore et al. [26] investigated, via an adapted mathematical model, the differences in transient and endemic behavior of chikungunya and dengue, risk of emergence for different virus-vector assemblages, and the role that virus evolution plays in disease dynamics and risk. Poletti et al. [29] developed a chikungunya transmission model for the spread of the epidemic in both humans and mosquitoes; the model involves a temporal dynamics of vector (*Aedes albopictus*), depending on climatic factors. In the study, they provided estimates of the transmission potential of the virus and assessed the efficacy of the measures undertaken by public health authorities to control the epidemic spread in Italy. Yakob and Clements [30] developed a simple, deterministic mathematical model for the transmission of the virus between humans and mosquitoes. They fitted the model to the large Reunion epidemic data and estimated the type reproduction number for chikungunya; their model provided a close approximation of both the peak incidence of the outbreak and the final epidemic size. Pongsumpun and Sangsawang [21] developed and studied theoretically an age-structured model for chikungunya involving juvenile and adult human populations, giving conditions for the disease-free and endemic states, respectively. They also suggested alternative way for controlling the disease.

The aim of this study is to develop a new deterministic transmission model to gain qualitative insight into the effects of age on chikungunya transmission dynamics and to determine the importance or otherwise of the inclusion of age in the transmission dynamics. A notable feature of the model is the incorporation of three different human age classes involving juvenile, adult, and senior human populations; the model also involves two infectious human classes, notably the asymptomatic and symptomatic classes. The paper is organized as follows; the model is formulated in Section 2, the analysis of the mathematical properties of the model is stated in Section 3. The effect of the age structure on the disease transmission is explored in Section 4. The sensitivity analysis of the model is investigated in Section 4.1. Following the result obtained from the sensitivity analysis, various control strategies are implemented in Section 5. The key theoretical and epidemiological results from this study are discussed and summarized in Section 6.

## 2. Model Formulation

The model is formulated as follows with human and mosquito subgroups. The human population is divided into juvenile, adult, and senior subpopulations. The human subgroup is further divided into susceptible (*S*_*i*_), exposed (*E*_*i*_), symptomatic (*I*_*Si*_), asymptomatic (*I*_*Ai*_), and recovered (*R*_*i*_), where *i* = *J*, *A*, *S* for the juvenile, adult, and senior subpopulations. Thus, the total human population *N*_*H*_(*t*) = *S*_*J*_(*t*) + *E*_*J*_(*t*) + *I*_*AJ*_(*t*) + *I*_*SJ*_(*t*) + *R*_*J*_(*t*) + *S*_*A*_(*t*) + *E*_*A*_(*t*) + *I*_*AA*_(*t*) + *I*_*SA*_(*t*) + *R*_*A*_(*t*) + *S*_*S*_(*t*) + *E*_*S*_(*t*) + *I*_*AS*_(*t*) + *I*_*SS*_(*t*) + *R*_*S*_(*t*). The mosquito population is divided into three classes consisting of susceptible mosquitoes (*S*_*M*_), exposed mosquitoes (*E*_*M*_), and infected mosquitoes (*I*_*M*_). Hence, the total mosquito population *N*_*M*_(*t*) = *S*_*M*_(*t*) + *E*_*M*_(*t*) + *R*_*M*_(*t*).

Individuals move from one class to the other as their status evolves with respect to the disease. The population of susceptible juvenile (*S*_*J*_) is generated at the rate *π*_*J*_ via birth or immigration. It is assumed that there is no vertical transmission or immigration of infectious humans; thus there is no inflow into the infectious classes. The population is reduced by the juvenile maturation at the rate *α* and by natural death at the rate *μ*_*J*_. The infection rate of susceptible juveniles *λ*_*J*_ is given as

(1) $$\begin{array}{c} \lambda_{J}=\frac{\beta_{J}b_{M}I_{M}}{N_{H}}. \end{array}$$

The parameter *β*_*J*_ in (1) is the probability that a bite from an infectious mosquito leads to infection of the susceptible juvenile and the parameter *b*_*M*_ is the mosquito biting rate. The derivation of (1) is given in Appendix A.

Similarly, it can be shown that the rate at which mosquitoes acquire infection from infectious (asymptomatic and symptomatic) human hosts is given by

(2) $$\begin{array}{c} \lambda_{M}=\frac{\beta_{M}b_{M}\left(I_{AJ}+I_{SJ}+I_{AA}+I_{SA}+I_{AS}+I_{SS}\right)}{N_{H}}. \end{array}$$

The parameter *β*_*M*_ is the probability that a bite from a susceptible mosquito to a human leads to infection of the mosquito.

Susceptible juveniles are infected by the chikungunya virus at a rate *λ*_*J*_ and move into the exposed class. Thus, the susceptible population is given as

(3) $$\begin{array}{c} \frac{dS_{J}}{dt}=\pi_{J}-\frac{\beta_{J}b_{M}S_{J}I_{M}}{N_{H}}-\alpha S_{J}-\mu_{J}S_{J}. \end{array}$$

The exposed juvenile population is generated following infection of the susceptible juveniles by infected mosquitoes. A fraction (1 − *ε*_*J*_) of exposed juveniles enter the asymptomatic class *I*_*AJ*_(*t*) at the rate (1 − *ε*_*J*_)*σ*_*J*_ and the remaining fraction (*ε*_*J*_) goes into the symptomatic class (*I*_*AS*_) at the rate *ε*_*J*_ *σ*_*J*_. The population of the exposed juvenile is reduced by the juvenile maturation at the rate *α*. The exposed juvenile population is further reduced by natural death at the rate *μ*_*J*_. Thus, the exposed population is given as

(4) $$\begin{array}{c} \frac{dE_{J}}{dt}=\frac{\beta_{J}b_{M}S_{J}I_{M}}{N_{H}}-\alpha_{J}E_{J}-\left(\;\sigma_{J}+\mu_{J}\;\right)E_{J}. \end{array}$$

Members of the juvenile asymptomatic class *I*_*AJ*_(*t*) are generated from the fraction that moved from the juvenile exposed class. This class is reduced by maturation to the adult asymptomatic class (*I*_*AA*_) at the rate *α*, by recovery (either naturally or via the use of treatment) at a rate *γ*_*AJ*_ to the recovered class. Similarly, members of the juvenile symptomatic class *I*_*SJ*_(*t*) are populated from the fraction that moved from the juvenile exposed class. The class is reduced due to maturation to the adult symptomatic class (*I*_*SA*_) at the rate *α* and by progression to the recovered class recovery at a rate *γ*_*SJ*_. These populations are further reduced by natural death at the rate *μ*_*J*_; chikungunya rarely results in death [1, 2]; as such we have ignored the disease induced death rate. Thus, the equations for these classes are given as follows:

(5) dIAJdt=εJσJEJ−αIAJ−γAJ+μJIAJ,dISJdt=1−εJσJEJ−αISJ−γSJ+μJISJ.

The juvenile recovered class *R*_*J*_ is populated from the juvenile asymptomatic and symptomatic classes; the class is reduced by maturation to the adult class at a rate *α* and by natural death at a rate *μ*_*J*_. The equation for this class is given as follows:

(6) $$\begin{array}{c} \frac{dR_{J}}{dt}=\gamma_{AJ}I_{AJ}+\gamma_{SJ}I_{SJ}-\alpha R_{J}-\mu_{J}R_{J}. \end{array}$$

The corresponding equations (susceptible, exposed, asymptomatic, symptomatic, and recovered) for the adult and senior classes are similarly obtained; additionally there is a maturation rate *ξ* from the adult population into the senior class. We assume that the recovery rates from adults asymptomatic and symptomatic classes are greater than those from juvenile classes which in turn are greater than those from the senior classes (i.e., *γ*_*AA*_, *γ*_*SA*_ > *γ*_*AJ*_, *γ*_*SJ*_ > *γ*_*AS*_, *γ*_*SS*_) [36]. Furthermore, we assume that seniors progress more quickly to the asymptomatic and symptomatic classes than juveniles and adults (i.e., *σ*_*S*_ > *σ*_*J*_, *σ*_*A*_) [36].

The population of the susceptible mosquitoes (*S*_*M*_) is generated by the recruitment rate *π*_*M*_ and reduced following effective contact with an infected human. All mosquitoes classes are reduced by natural death at a rate *μ*_*M*_. The equation for this class is given as follows:

(7) dSMdt=πM−βMbMIAJ+ISJ+IAA+ISA+IAS+ISSNHSM−μMSM.

Mosquitoes in the exposed class *E*_*M*_ are generated following the infection of the susceptible mosquitoes. They progress to the infected class at a rate *σ*_*M*_. The equation for the exposed mosquitoes dynamics is given as follows:

(8) dEMdt=βMbMIAJ+ISJ+IAA+ISA+IAS+ISSNHSM−μMSM−σMEM.

The infected mosquitoes class are populated from the exposed mosquitoes. The equation for this class is given as follows:

(9) $$\begin{array}{c} \frac{dI_{M}}{dt}=\sigma_{M}E_{M}-\mu_{M}I_{M}. \end{array}$$

Combining the aforementioned derivations and assumptions the model for the transmission dynamics of chikungunya virus in a population is given by the following deterministic system of nonlinear differential equations:

(10) dSJdt=πJ−βJbMSJIMNH−αSJ−μJSJ,dEJdt=βJbMSJIMNH−αEJ−σJ+μJEJ,dIAJdt=εJσJEJ−αIAJ−γAJ+μJIAJ,dISJdt=1−εJσJEJ−αISJ−γSJ+μJISJ,dRJdt=γAJIAJ+γSJISJ−αRJ−μJRJ,dSAdt=αSJ−βAbMSAIMNH−ξSA−μASA,dEAdt=αEJ+βAbMSAIMNH−ξEA−σA+μAEA,dIAAdt=αIAJ+εAσAEA−ξIAA−γAA+μAIAA,dISAdt=αISJ+1−εAσAEA−ξISA−γSA+μAISA,dRAdt=αRJ+γAAIAA+γSAISA−ξRA−μARA,dSSdt=ξSA−βSbMSSIMNH−μSSS,dESdt=ξEA+βSbMSSIMNH−σS+μSES,dIASdt=ξIAA+εSσSES−γAS+μSIAS,dISSdt=ξISA+1−εSσSES−γSS+μSISS,dRSdt=ξRA+γASIAS+γSSISS−μSRS,dSMdt=πM−βMbMIAJ+ISJ+IAA+ISA+IAS+ISSNHSM−μMSM,dEMdt=βMbMIAJ+ISJ+IAA+ISA+IAS+ISSNHSM−σM+μMEM,dIMdt=σMEM−μMIM.

**Figure 1:**
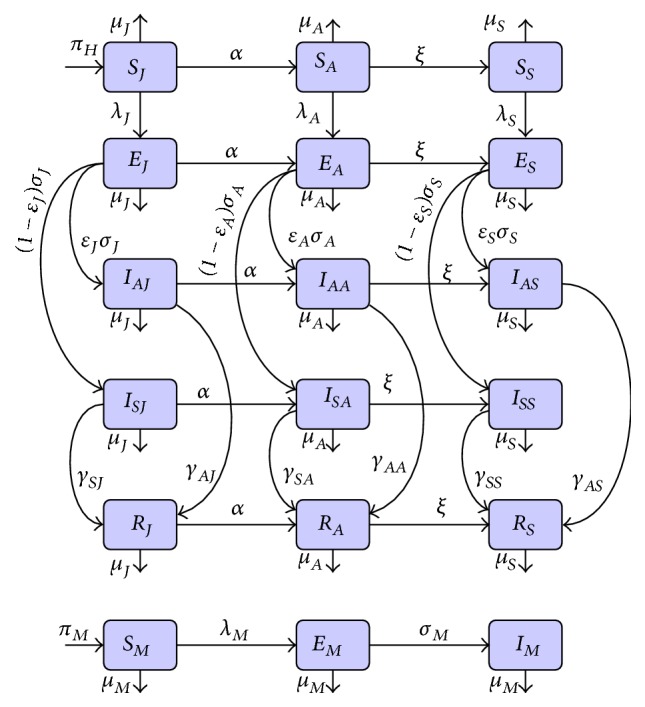
Systematic flow diagram of the age-structured chikungunya model (10).

The flow diagram of the age-structured chikungunya model (10) is depicted in Figure 1 and the associated variables and parameters are described in Table 1. Model (10) is an extension of some of the chikungunya transmission models (e.g., those in [21, 25–30]) by (*inter alia*):

- Including a compartment for the exposed humans and mosquitoes (this was not considered in [21, 27, 28]).
- Adding a compartment for asymptomatic and symptomatic individuals (these were not considered in [25, 26, 30]).
- Including an age structure for humans (this was not included in [26, 29]).
- Adding compartments for seniors (these were not included in [21, 25–28, 30]).

## 3. Analysis of the Model

### 3.1. Basic Qualitative Properties

#### 3.1.1. Positivity and Boundedness of Solutions

For the age-structured chikungunya transmission model (10) to be epidemiologically meaningful, it is important to prove that all its state variables are nonnegative for all time. In other words, solutions of the model system (10) with non-negative initial data will remain non-negative for all time *t* > 0.

**Lemma 1 .**

Let the initial data *F*(0) ≥ 0, where *F*(*t*) = (*S*_*J*_, *E*_*J*_, *I*_*AJ*_, *I*_*SJ*_, *R*_*J*_, *S*_*A*_, *E*_*A*_, *I*_*AA*_, *I*_*SA*_, *R*_*A*_, *S*_*S*_, *E*_*S*_, *I*_*AS*_, *I*_*SS*_, *R*_*A*_, *S*_*M*_, *E*_*M*_, *I*_*M*_). Then the solutions *F*(*t*) of the age-structured chikungunya model (10) are nonnegative for all *t* > 0. Furthermore

(11) limsupt→∞⁡ NHt=πJμH,limsupt→∞⁡ NMt=πMμM

with

(12) NHt=SJt+EJt+IAJt+ISJt+RJt+SAt+EAt+IAAt+ISAt+RAt+SSt+ESt+IASt+ISSt+RSt,NMt=SMt+EMt+IMt.

The proof of Lemma 1 is given in Appendix B.

#### 3.1.2. Invariant Regions

The age-structured chikungunya model (10) will be analyzed in a biologically feasible region as follows. Consider the feasible region

(13) $$\begin{array}{c} \Omega =\Omega_{H}\times \Omega_{M}\subset R_{+}^{15}\times R_{+}^{3} \end{array}$$

with

(14) ΩH=SJ,EJ,ISJ,IAJ,RJ,SA,EA,ISA,IAA,RA,SS,ES,ISS,IAS,RS:NHt≤πJμH,ΩM=SM,EM,IM:NMt≤πMμM.

**Lemma 2 .**

The region *Ω* ⊂ *ℝ*_+_^18^ is positively invariant for the age-structured chikungunya model (10) with nonnegative initial conditions in *ℝ*_+_^18^.

The proof of Lemma 2 is given in Appendix C.

In the next section, the conditions for the existence and stability of the equilibria of the age-structured chikungunya model (10) are explored.

### 3.2. Stability of Disease-Free Equilibrium (DFE)

The age-structured chikungunya model (10) has a disease-free equilibrium (DFE), obtained by setting the right-hand sides of the equations in the model to zero, given by

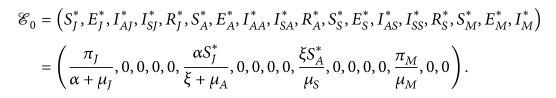

(15)
The linear stability of *ℰ*_0_ can be established using the next generation operator method on system (10). Taking *E*_*J*_, *I*_*SJ*_, *I*_*AJ*_, *E*_*A*_, *I*_*SA*_, *I*_*AA*_, *E*_*S*_, *I*_*SS*_, *I*_*AS*_, *E*_*M*_, *I*_*M*_ as the infected compartments and then using the notation in [42], the Jacobian matrices *F* and *V* for the new infection terms and the remaining transfer terms are, respectively, given by

(16) F=0000000000ΦJ00000000000000000000000000000000ΦA00000000000000000000000000000000ΦS00000000000000000000000ΦMΦM0ΦMΦM0ΦMΦM0000000000000,

where Φ_*J*_ = *β*_*J*_ *b*_*M*_ *μ*_*H*_/*k*_1_, Φ_*A*_ = *β*_*A*_ *b*_*M*_ *α*/*k*_1_, Φ_*S*_ = *β*_*S*_ *b*_*M*_ *ξα*/*μ*_*H*_ *k*_1_, Φ_*M*_ = *π*_*M*_ *β*_*M*_ *b*_*M*_ *μ*_*H*_/*μ*_*M*_ *π*_*J*_, and

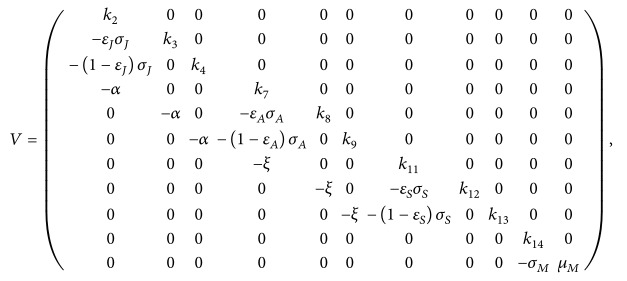

(17)
where *k*_1_ = *π*_*J*_, *k*_2_ = *α* + *σ*_*J*_ + *μ*_*J*_, *k*_3_ = *α* + *γ*_*AJ*_ + *μ*_*J*_, *k*_4_ = *α* + *γ*_*SJ*_ + *μ*_*J*_, *k*_7_ = *ξ* + *σ*_*A*_ + *μ*_*A*_, *k*_8_ = *ξ* + *γ*_*AA*_ + *μ*_*A*_, *k*_9_ = *ξ* + *γ*_*SA*_ + *μ*_*A*_, *k*_11_ = *σ*_*S*_ + *μ*_*S*_, *k*_12_ = *γ*_*AS*_ + *μ*_*S*_, *k*_13_ = *γ*_*SS*_ + *μ*_*S*_, and *k*_14_ = *σ*_*M*_ + *μ*_*M*_.

It follows that the reproduction number of the age-structured chikungunya model (10) is given by

(18) $$\begin{array}{c} R_{0}=\rho \left(\;FV^{-1}\;\right)=\sqrt{\left(R_{JM}+R_{AM}+R_{SM}\right)R_{MH}}, \end{array}$$

where *ρ* is the spectral radius and

(19) RJM=βJbMSJ∗k11k12k13k7k8k9σJ1−εJk3+εJk4+αk11k12k13σJk71−εJk3k8+εJk9k4+σAk4k31−εAk8+εAk9+ξασJk11k71−εJk3k8k12+εJk4k9k13+σAk3k4k111−εAk8k12+εAk9k13+σSk3k4k8k91−εSk12+εSk13,RAM=βAbMSA∗k2k3k4σAk12k11k131−εAk8+εAk9+ξσAk111−εAk8k12+εAk9k13+σSk8k91−εSk12+εSk13,RSM=βSbMσSSS∗k2k3k4k8k7k91−εSk12+k13εS,RMH=σMβMSM∗μH2bMk2k3k4k8k7k9k11k12k13k14μMπJ2.

Furthermore, the expression *ℛ*_*JM*_ is the number of secondary infections in juveniles by one infectious mosquito, *ℛ*_*AM*_ is the number of secondary infections in adults by one introduced infectious mosquito, *ℛ*_*SM*_ is the number of secondary infections in seniors as a result of one infectious mosquito, and lastly *ℛ*_*MH*_ is the number of secondary infections in mosquitoes resulting from a newly introduced infectious juvenile, adult, and senior. Further, using Theorem 2 in [42], the following result is established.

**Lemma 3 .**

The disease-free equilibrium (DFE) of the age-structured chikungunya model (10) is locally asymptotically stable (LAS) if *ℛ*_0_ < 1 and unstable if *ℛ*_0_ > 1.

**Figure 2:**
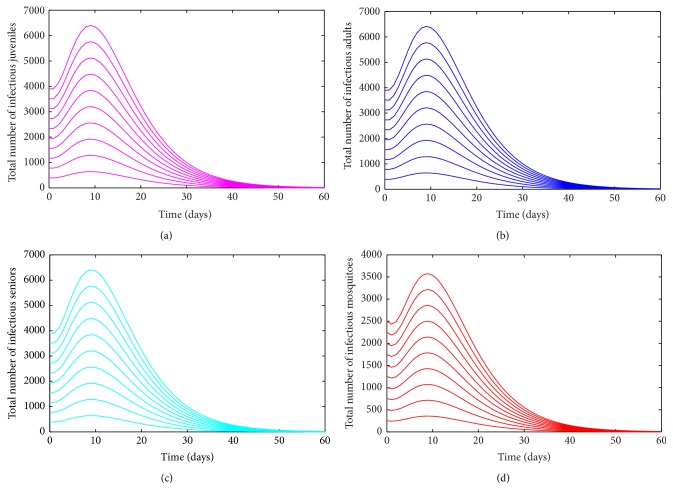
Simulation of the age-structured chikungunya model (10) as a function of time when *ℛ*_0_ < 1. (a) Total number of infectious (asymptomatic and symptomatic) juveniles. (b) Total number of infectious (asymptomatic and symptomatic) adults. (c) Total number of infectious (asymptomatic and symptomatic) seniors. (d) Total number of infectious mosquitoes. Parameter values used are as given in Table 3.

The basic reproduction number *ℛ*_0_ is defined as the average number of new infections that result from one infectious individual in a population that is fully susceptible [42–45]. The epidemiological significance of Lemma 3 is that chikungunya will be eliminated from the community if the reproduction number (*ℛ*_0_) can be brought to (and maintained at) a value less than unity.  Figure 2 shows convergence of the solutions of the age-structured chikungunya model (10) to the DFE (*ℰ*_0_) for the case when *ℛ*_0_ < 1 (in accordance with Lemma 3).

### 3.3. Global Asymptotic Stability of the DFE

Consider the feasible region

(20) $$\begin{array}{c} \Omega_{1}=\left\{\;X\in \Omega :S_{J}\leq S_{J}^{*},S_{A}\leq S_{A}^{*},S_{S}\leq S_{S}^{*},S_{M}\leq S_{M}^{*}\;\right\}, \end{array}$$

where *X* = *S*_*J*_, *E*_*J*_, *I*_*SJ*_, *I*_*AJ*_, *R*_*J*_, *S*_*A*_, *E*_*A*_, *I*_*SA*_, *I*_*AA*_, *R*_*A*_, *S*_*S*_, *E*_*S*_, *I*_*SS*_, *I*_*AS*_, *R*_*S*_, *S*_*M*_, *E*_*M*_, *I*_*M*_.

**Lemma 4 .**

The region *Ω*_1_ is positively invariant for the age-structured chikungunya model (10).

The proof of Lemma 4 is given in Appendix D.

**Theorem 5 .**

The DFE, *ℰ*_0_, of the age-structured chikungunya model (10), is globally asymptotically stable (GAS) in *Ω*_1_ whenever *ℛ*_0_ ≤ 1.

The proof of Theorem 5 is given in Appendix E.

### 3.4. Existence of Endemic Equilibrium Point (EEP)

In this section, we will investigate conditions for the existence of endemic equilibria (i.e., equilibria where the infected components of the age-structured model (10) are nonzero).

Let

(21) E1=SJ∗∗,EJ∗∗,IAJ∗∗,ISJ∗∗,RJ∗∗,SA∗∗,EA∗∗,IAA∗∗,ISA∗∗,RA∗∗,SS∗∗,EA∗∗,IAS∗∗,ISS∗∗,RS∗∗,SM∗∗,EM∗∗,IM∗∗

be an arbitrary endemic equilibrium of age-structured chikungunya model (10). Also, let

(22) λJ∗∗=βJbMIM∗∗NH∗∗,λA∗∗=βAbMIM∗∗NH∗∗,λS∗∗=βSbMIM∗∗NH∗∗,λM∗∗=βMbMIAJ∗∗+ISJ∗∗+IAA∗∗+ISA∗∗+IAS∗∗+ISS∗∗NH∗∗

be the forces of infection for susceptible juveniles, adults, and seniors and susceptible mosquitoes at steady state, respectively. Components of the steady-state solution of the equations of the age-structured chikungunya model (10) are given in Appendix F. Substituting the expressions for *I*_*AJ*_^*∗∗*^, *I*_*SJ*_^*∗∗*^, *I*_*AA*_^*∗∗*^, *I*_*SA*_^*∗∗*^, *I*_*AS*_^*∗∗*^ and *I*_*SS*_^*∗∗*^ into (22) for *λ*_*M*_^*∗∗*^ and simplifying gives

(23) $$\begin{array}{c} \lambda_{M}^{**}=\frac{b_{M}^{2}\beta_{M}I_{M}^{**}\mu_{H}\left[a_{2}{\left(I_{M}^{**}\right)}^{2}+a_{1}I_{M}^{**}+a_{0}\right]}{k_{2}k_{3}k_{4}k_{7}k_{8}k_{9}k_{11}k_{12}\left(\beta_{J}b_{M}I_{M}^{**}\mu_{H}+k_{1}\pi_{J}\right)\left(\beta_{A}b_{M}I_{M}^{**}\mu_{H}+\pi_{J}k_{6}\right)\left(\beta_{S}b_{M}I_{M}^{**}\mu_{H}+\pi_{J}\mu_{S}\right)k_{13}}, \end{array}$$

where

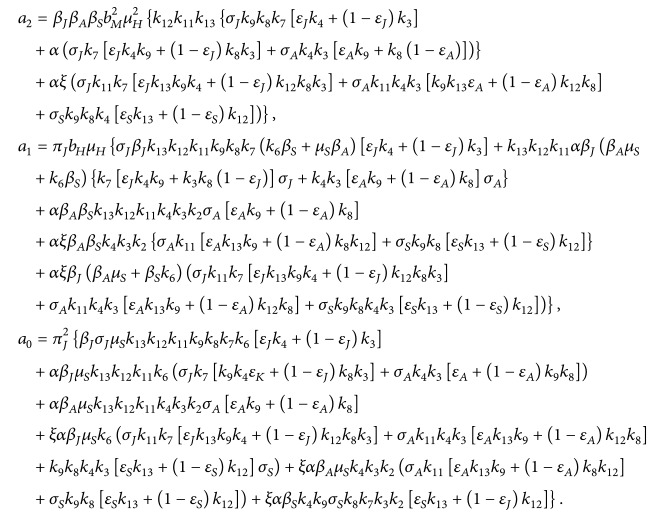

(24)
Substituting expression for *I*_*M*_^*∗∗*^ into the force of infection *λ*_*J*_^*∗∗*^ in (22) gives

(25) $$\begin{array}{c} \lambda_{J}^{**}=\frac{\beta_{J}b_{M}\sigma_{M}\lambda_{M}^{**}\pi_{M}\mu_{H}}{k_{14}\mu_{M}\pi_{J}\left(\lambda_{M}^{**}+\mu_{M}\right)} \end{array}$$

and then solving for *λ*_*M*_^*∗∗*^ gives

(26) $$\begin{array}{c} \lambda_{M}^{**}=-\frac{\lambda_{J}^{**}k_{14}\mu_{M}^{2}\pi_{J}}{\left(\lambda_{J}^{**}k_{14}\mu_{M}\pi_{J}-\beta_{J}b_{M}\sigma_{M}\pi_{M}\mu_{H}\right)}. \end{array}$$

Substituting this result into (23), and simplifying, leads to the following cubic equation:

(27) $$\begin{array}{c} b_{3}{\left(\;\lambda_{J}^{**}\;\right)}^{3}+b_{2}{\left(\;\lambda_{J}^{**}\;\right)}^{2}+b_{1}\lambda_{J}^{**}+b_{0}\left(\;1-R_{0}^{2}\;\right), \end{array}$$

where

(28) b3=πJ4βJβAβSbMμHμM2k2k3k4k8k7k9k11k12k13k14+πJ3βMμMk14a2,b2=πJ4βJβAβSbMμHμM2k1k2k3k4k8k7k9k11k12k13k14−πJ2βJπMβMbMσMμHa2+πJ2βJμHβMμMbMk14a1+πJ4βJ2βAbMμSμH2μM2k2k3k4k8k7k9k12k11k13k14+πJ4βJ2βSμHbMμM2k2k3k4k6k7k8k9k11k12k13k14,b1=πJβJ2μH2βMbM2μMk14a0+πJ4βJ2βAbMμSμH2μM2k1k2k3k4k8k7k9k11k12k13k14−πJβJ2bM2πMβMσMμH2a1+πJ4βJ3bMμSμH2μM2k2k3k4k6k7k8k9k11k12k13k14+πJ4βJ2βSbMμHμM2k1k2k3k4k6k7k8k9k11k12k13k14,b0=πJ4βJ3bMμH2μSμM2k1k2k3k4k6k8k7k9k11k12k13k14.

Thus, the number of possible positive real roots polynomial (27) can have depends on the signs of *b*_2_ and *b*_1_. This can be analyzed using the Descartes Rule of Signs on the cubic polynomial *f*(*x*) = *c*_3_ *x* ^3^ + *c*_2_ *x* ^2^ + *c*_1_ *x* + *c*_0_, given in (27) (with *x* = *λ*_*J*_^*∗∗*^, *c*_3_ = *b*_3_, *c*_2_ = *b*_2_, *c*_1_ = *b*_1_, *c*_0_ = *b*_0_(1 − *ℛ*_0_^2^)). The various possibilities for the roots of *f*(*x*) are tabulated in Table 2.

The following results (Theorem 6) follow from the various possible combinations for the roots of *f*(*x*), in Table 2.

**Theorem 6 .**

The age-structured chikungunya model (10) has a unique endemic equilibrium if *ℛ*_0_ > 1.

**Figure 3:**
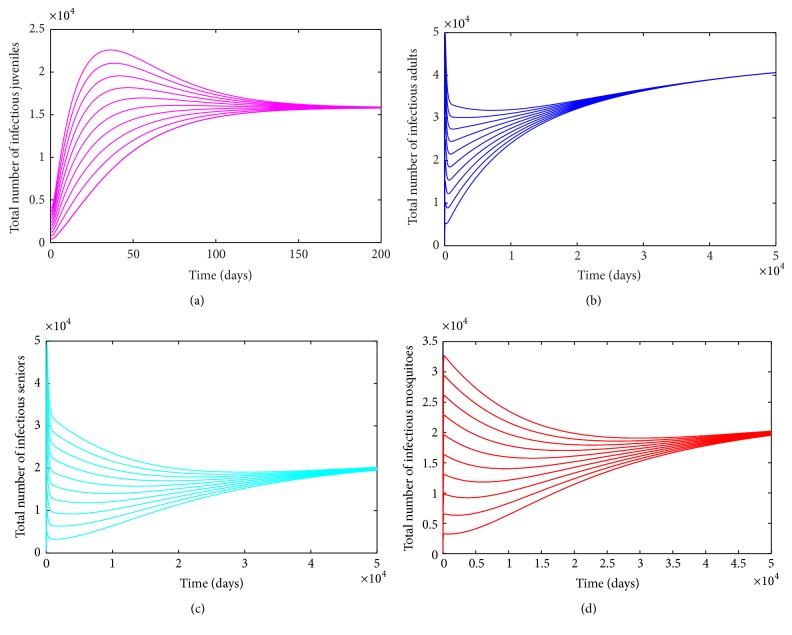
Simulation of the age-structured chikungunya model (10) as a function of time when *ℛ*_0_ > 1. (a) Total number of infectious (asymptomatic and symptomatic) juveniles. (b) Total number of infectious (asymptomatic and symptomatic) adults. (c) Total number of infectious (asymptomatic and symptomatic) seniors. (d) Total number of infectious mosquitoes. Parameter values used are as given in Table 3.

Numerical simulations of the age-structured chikungunya model (10), depicted in Figure 3, show convergence to a unique endemic equilibrium when *ℛ*_0_ > 1 (suggesting that the unique EEP of the age-structured chikungunya model (10) is asymptotically stable when it exists).

### 3.5. Backward Bifurcation Analysis: Special Case

Chikungunya rarely leads to death of the infected individuals [1, 2]; however a number of deaths have been reported as a result of the infection [3, 5, 30, 46, 47]. We introduced into the asymptomatic and symptomatic human compartments disease-induced mortality parameters (*δ*_*AJ*_, *δ*_*SJ*_, *δ*_*AA*_, *δ*_*SA*_, *δ*_*AS*_, *δ*_*SS*_) and study the implication on the dynamics of the disease transmission. Thus, the asymptomatic and symptomatic juveniles, adults, and seniors compartments of age-structured chikungunya model (10) can be written as follows:

(29) dIAJdt=εJσJEJ−αIAJ−γAJ+μH+δAJIAJ,dISJdt=1−εJσJEJ−αISJ−γSJ+μH+δSJISJ,dIAAdt=αIAJ+εAσAEA−ξIAA−γAA+μH+δAAIAA,dISAdt=αISJ+1−εAσAEA−ξISA−γSA+μH+δSAISA,dIASdt=ξIAA+εSσSES−γAS+μH+δASIAS,dISSdt=ξISA+1−εSσSES−γSS+μH+δSSISS.

It can be shown that the reproduction number for the age-structured chikungunya model (10) with the asymptomatic and symptomatic human compartments stated in (29) is given by

(30) R~0R0δAJ,δSJ,δAA,δSA,δAS,δSS≠0=R~JM+R~AM+R~SMR~MH,

where

(31) R~JM=βJbMSJ∗k11k12k13σJk7k8k91−εJk3+εJk4+αk11k12k13σJk71−εJk3k8+εJk9k4+σAk4k31−εAk8+εAk9+ξασJk11k71−εJk3k8k12+εJk4k9k13+σAk3k4k111−εAk8k12+εAk9k13+σSk3k4k8k91−εSk12+εSk13,R~AM=βAbMSA∗k2k3k4σAk12k11k131−εAk8+εAk9+ξσAk111−εAk8k12+εAk9k13+σSk8k91−εSk12+εSk13,R~SM=βSbMσSSS∗k2k3k4k8k7k91−εSk12+k13εS,R~MH=σMβMSM∗μH2bMk2k3k4k8k7k9k11k12k13k14μMπJ2,

with *k*_3_ = *α* + *γ*_*AJ*_ + *μ*_*J*_ + *δ*_*SJ*_, *k*_4_ = *α* + *γ*_*SJ*_ + *μ*_*J*_ + *δ*_*SJ*_, *k*_8_ = *ξ* + *γ*_*AA*_ + *μ*_*A*_ + *δ*_*AA*_, *k*_9_ = *ξ* + *γ*_*SA*_ + *μ*_*A*_ + *δ*_*SA*_, *k*_12_ = *γ*_*AS*_ + *μ*_*S*_ + *δ*_*AS*_, and *k*_13_ = *γ*_*SA*_ + *μ*_*S*_ + *δ*_*SA*_.

Models of disease transmission typically undergo a simple transcritical bifurcation (exchange of stability from the DFE to an endemic equilibrium) at ${\tilde{ℛ}}_{0}=1$ . The age-structured chikungunya model (10) with the asymptomatic and symptomatic human compartments stated in (29) is investigated for the possibility of the phenomenon of backward bifurcation (where a stable DFE coexists with a stable endemic equilibrium when the reproduction number, ${\tilde{ℛ}}_{0}$ , is less than unity) [48–57]. The epidemiological implication of backward bifurcation is that the effective control (or elimination) of chikungunya virus in the system is dependent on the initial sizes of the subpopulations. The possibility of the phenomenon of backward bifurcation in the age-structured chikungunya model (10) with the asymptomatic and symptomatic human compartments stated in (29) is explored using the centre manifold theory [51], as described in [58, Theorem 4.1].

**Theorem 7 .**

The age-structured chikungunya model (10) with the asymptomatic and symptomatic human compartments stated in (29) undergoes backward bifurcation at ${\tilde{ℛ}}_{0}=1$ whenever inequality (G.9), given in Appendix G, holds.

**Figure 4:**
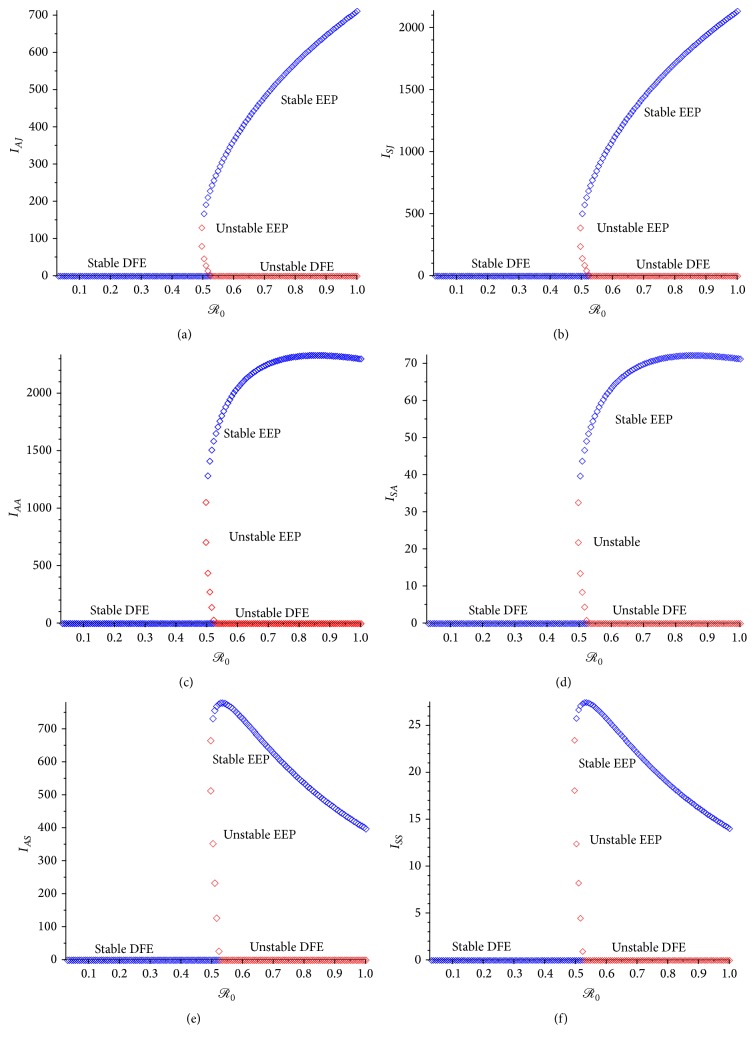
Backward bifurcation plot of the age-structured model (10) with the asymptomatic and symptomatic human compartments given in (29). (a) Asymptomatic juvenile; (b) symptomatic juvenile. (c) Asymptomatic adult; (d) symptomatic adult. (e) Asymptomatic senior; (f) symptomatic senior. Parameter values used are as given in Table 3.

The proof of Theorem 7 is given in Appendix G. The backward bifurcation property of the age-structured chikungunya model (10) with the asymptomatic and symptomatic human compartments given in (29) is illustrated by simulating the model using a set of parameter values given in Table 3 (such that the bifurcation parameters, *a* and *b*, given in Appendix G, take the values *a* = 0.001792 and *b* = 0.09056, resp.). The backward bifurcation phenomenon of the age-structured chikungunya model (10) with the asymptomatic and symptomatic human compartments stated in (29) makes the effective control of the chikungunya in the population difficult, since in this case, disease control when ${\tilde{ℛ}}_{0}<1$ is dependent on the initial sizes of the subpopulations of the age-structured chikungunya model (10) with the asymptomatic and symptomatic human compartments stated in (29). This phenomenon is illustrated numerically in Figure 4.

## 4. Effect of Age Structure

Following the approach in [59], the effect of age structure on the dynamics of the age-structured chikungunya model (10) will now be investigated by comparing its dynamical behavior with those of an equivalent model with no age structure given by

(32) dSHdt=πH−βHbMIMNHSH−αSH−μHSH,dEHdt=βHbMIMNHSH−αEH−σH+μHEH,dIAHdt=εHσHEH−αIAH−γAH+μHIAH,dISHdt=1−εHσHEH−αISH−γSH+μHISH,dRHdt=γAHIAH+γSHISH−αRH−μHRH,dSMdt=πM−βMbMIAH+ISHNHSM−μMSM,dEMdt=βMbMIAH+ISHNHSM−σM+μMEM,dIMdt=σMEM−μMIM,

where *S*_*H*_ = *S*_*J*_ + *S*_*A*_ + *S*_*S*_, *E*_*H*_ = *E*_*J*_ + *E*_*A*_ + *E*_*S*_, *I*_*AH*_ = *I*_*AJ*_ + *I*_*AA*_ + *I*_*AS*_, *I*_*SH*_ = *I*_*SJ*_ + *I*_*SA*_ + *I*_*SS*_, *R*_*H*_ = *R*_*J*_ + *R*_*A*_ + *R*_*S*_ and *σ*_*H*_ = *σ*_*J*_ + *σ*_*A*_ + *σ*_*S*_, *γ*_*AH*_ = *γ*_*AJ*_ + *γ*_*AA*_ + *γ*_*AS*_, *γ*_*SH*_ = *γ*_*SJ*_ + *γ*_*SA*_ + *γ*_*SS*_, and *α* = *ξ* = 0.

The DFE of model (32) is given by

(33) E01SH∗,EH∗,IAH∗,ISH∗,RH∗,SM∗,EM∗,IM∗=πHμH,0,0,0,0,πMμM,0,0

and the associated reproduction number is

(34) RH≔πMβMσMπHβHbM2g4εHσH+g31−εHσHg2g3g4g6μM2μH,

where *g*_1_ = *μ*_*H*_, *g*_2_ = *σ*_*H*_ + *μ*_*H*_, *g*_3_ = *γ*_*AH*_ + *μ*_*H*_, *g*_4_ = *γ*_*SH*_ + *μ*_*H*_, *g*_5_ = *μ*_*H*_, and *g*_6_ = *σ*_*M*_ + *μ*_*M*_.

Using the approach in Section 3.3, we can show that the reduced model (32) has a DFE that is GAS if *δ*_*AH*_ = *δ*_*SH*_ = 0, whenever *ℛ*_*H*_ < 1; as such model (32) will not undergo backward bifurcation since the bifurcation coefficient, *a*, is given by

(35) $$\begin{array}{c} a=\frac{2b_{M}\mu_{H}\left(v_{2}w_{1}w_{8}\beta_{H}+v_{7}w_{3}w_{6}\beta_{M}+v_{7}w_{4}w_{6}\beta_{M}\right)}{\pi_{H}}; \end{array}$$

using the expression in (38) below, (35) becomes

(36) $$\begin{array}{c} a=-\frac{2b_{M}^{5}\mu_{H}^{2}\sigma_{H}^{2}\pi_{M}^{2}\beta_{M}^{3}\sigma_{M}{\left[\varepsilon_{H}g_{4}+\left(1-\varepsilon_{H}\right)g_{3}\right]}^{2}\beta_{H}^{2}\left\{g_{3}g_{4}\mu_{M}g_{2}+b_{M}\beta_{M}\sigma_{H}g_{1}\left[\varepsilon_{H}g_{4}+\left(1-\varepsilon_{H}\right)g_{3}\right]\right\}}{\mu_{M}^{4}g_{6}\pi_{H}^{3}g_{3}^{3}g_{4}^{3}g_{2}^{3}}, \end{array}$$

where *g*_1_ = *μ*_*H*_, *g*_2_ = *σ*_*H*_ + *μ*_*H*_, *g*_3_ = *γ*_*AH*_ + *μ*_*H*_, and *g*_4_ = *γ*_*SH*_ + *μ*_*H*_.

However, if *δ*_*AH*_, *δ*_*SH*_ ≠ 0, then it follows that the reduced model (32) will undergo backward bifurcation if the coefficient *a*, in this case, given as

(37) $$\begin{array}{c} a=\frac{-2b_{M}\left[v_{2}w_{8}x_{1}\left(w_{2}+w_{3}+w_{5}\right)\beta_{H}+v_{7}w_{3}x_{6}\left(w_{3}+w_{1}+w_{2}+w_{5}\right)\beta_{M}-v_{7}w_{3}w_{6}x_{1}\beta_{M}\right]}{x_{1}^{2}}, \end{array}$$

is positive, where

(38) w1=−βHbMw8g1μM,w2=βHbMw8g2,w3=εHσHw2g3,w4=1−εHσHw2g4,w5=γAHw3+γSHw4g5,w6=−x6βMbMw3+w4μMx1,w7=x6βMbMw3+w4g6x1,w8>0,v1=0,v5=0,v6=0,v2=εHσHv3+1−εHσHv4g2,v3=1g3γAHv5+x6βMbMv7−v6x1,v4=1g4γSHv5+x6βMbMv7−v6x1,v7=σMv8g6,v8=βHbMv2μM

with *g*_1_ = *μ*_*H*_, *g*_2_ = *σ*_*H*_ + *μ*_*H*_, *g*_3_ = *γ*_*AH*_ + *μ*_*H*_ + *δ*_*AH*_, *g*_4_ = *γ*_*SH*_ + *μ*_*H*_ + *δ*_*SH*_, *g*_5_ = *μ*_*H*_, and

(39) $$\begin{array}{c} b=\frac{v_{7}x_{6}b_{M}w_{3}}{x_{1}}>0. \end{array}$$

**Figure 5:**
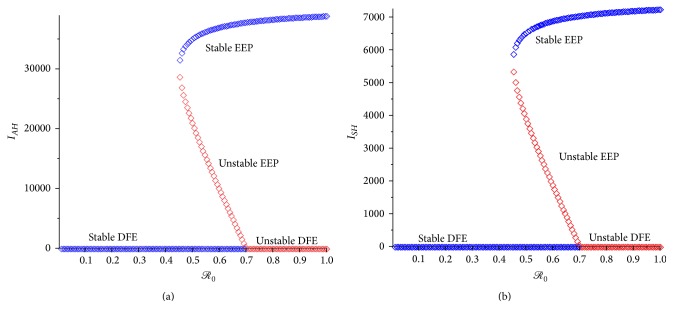
Backward bifurcation plot for without age-structured model (32). (a) Asymptomatic humans; (b) symptomatic humans. Parameter values used are as given in Table 3.

The phenomenon of backward bifurcation for model (32) without age structure is illustrated numerically in Figure 5.

Hence, the preceding analysis shows that the age-structured chikungunya model (10) and model (32) without age structure have the same qualitative dynamics with respect to the local and global asymptotic stability of the associated disease-free equilibrium (DFE) and the phenomenon of backward bifurcation.

Next, we compare the dynamics of the endemic equilibrium points of models (10) and (32). Let

(40) $$\begin{array}{c} E_{1}=\left(\;S_{H}^{**},E_{H}^{**},I_{SH}^{**},I_{AH}^{**},R_{H}^{**},S_{M}^{**},E_{M}^{**},I_{M}^{**}\;\right) \end{array}$$

be an arbitrary endemic equilibrium of the reduced model (29) and let

(41) λH∗∗=βHbMIM∗∗NH∗∗,λM∗∗=βMbMIAH∗∗+ISH∗∗NH∗∗

be the forces infection for susceptible humans and susceptible mosquitoes at steady state, respectively. Solving the equations of the reduced model (32) at steady state gives

(42) SH∗∗=πHλH∗∗+g1,EH∗∗=λH∗∗πHg2λH∗∗+g1,IAH∗∗=εHσHλH∗∗πHg3g2λH+g1,ISH∗∗=1−εHσHλH∗∗πHg4g2λH∗∗+g1,RH∗∗=σHλH∗∗πHγAHεHg4+γSH1−εHg3g5g4g2g3λH∗∗+g1,SM∗∗=πMλM∗∗+μM,EM∗∗=λMπMg6λM∗∗+μM,IM∗∗=σMλM∗∗πMg6μMλM∗∗+μM.

Substituting (42) into (41) shows that the positive endemic equilibrium of model (32) satisfies

(43) λH∗∗=πHg6μM2g2g3g4g1πHRH2−1πH2g6μMg2g3g4μM+bMβMμHεHσHg4+1−εHσHg3,

where ${ℛ}_{H}={\tilde{ℛ}}_{H}{|}_{\delta_{AH},\delta_{SH}\neq 0}$ . Hence, model (32) has a unique endemic equilibrium (obtained by substituting (42) into (43)) whenever ${\tilde{ℛ}}_{H}>1$ .

### 4.1. Sensitivity Analysis

**Figure 6:**
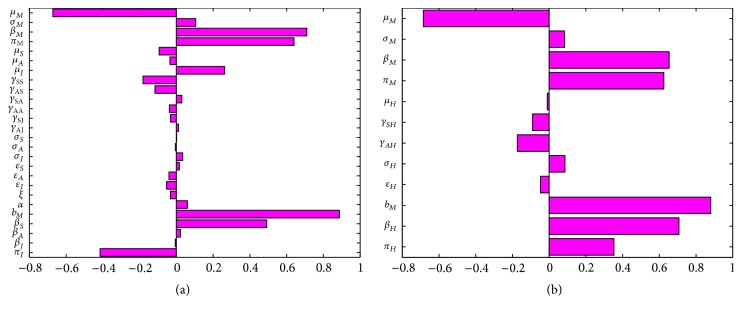
PRCC values for chikungunya models (10) and (32), using as response functions (a) the reproduction number *ℛ*_0_; (b) the reproduction number *ℛ*_*H*_. Parameter values (baseline) and ranges used are as given in Table 3.

Sensitivity analysis [60–62] is carried out, on the parameters of the age-structured chikungunya model (10), to determine which of the parameters have the most significant impact on the outcome of the numerical simulations of the model.  Figure 6(a) depicts the partial rank correlation coefficient (PRCC) values for each parameter of the models, using the ranges and baseline values tabulated in Table 3 (with the basic reproduction number, *ℛ*_0_, as the response function), from which it follows that the parameters that have the most influence on chikungunya transmission dynamics are the mosquito biting rate (*b*_*M*_), the transmission probability* per* contact in mosquitoes (*β*_*M*_) and in humans (*β*_*S*_), mosquito recruitment rate (*π*_*M*_), and the death rate of the mosquitoes (*μ*_*M*_). It is interesting to note that, from Figure 6(a), the transmission probability* per* contact in juvenile and adult (*β*_*J*_ and *β*_*A*_) is not as significant as that of the seniors. Thus, this study identifies the most important parameters that drive the transmission mechanism of the disease. The identification of these key parameters is vital to the formulation of effective control strategies for combating the spread of the disease. In other words, the results of this sensitivity analysis suggest that a strategy that reduces the mosquito biting rate (reduces *b*_*M*_), the mosquito recruitment rate (reduces *π*_*M*_), and the transmission probability* per* contact in mosquitoes (reduces *β*_*M*_) and in humans (reduces *β*_*S*_) and increases the death rate of the mosquito (increases *μ*_*M*_) will be effective in curtailing the spread of chikungunya virus in the community.

The sensitivity analysis was also carried out using model (32) without age-structured with *ℛ*_*H*_, as the response function. The dominant parameters in this case are *b*_*M*_, *β*_*M*_, *β*_*H*_, *π*_*M*_, and *μ*_*M*_. These results show that the same parameters are dominant for the two response functions (*ℛ*_0_ and *ℛ*_*H*_). This result further emphasizes the fact that the inclusion of age structure does not alter the dynamics of the transmission of the infection.

## 5. Assessment of Control Strategies

In order to reduce the number of infected human cases, values of some dominant parameters (the mosquito recruitment rate (*π*_*M*_), the death rate of the mosquito (*μ*_*M*_), and the transmission probability* per* contact in mosquitoes (*β*_*M*_) and in humans (*β*_*J*_, *β*_*A*_, and *β*_*S*_)) obtained from the sensitivity analysis were adjusted to capture mosquito-reduction strategy, personal-protection strategy, and the effect of the combination of both strategies (universal strategy). The reduction in the mosquito biting rate, a dominant parameter, is captured implicitly by the reduction of all the transmission probabilities. Three effectiveness levels (low, moderate, and high) were evaluated for each of the strategies using the initial conditions in Table 4 obtained from [19]. The numbers in Table 4 were estimated using the 1913 infected individuals obtained in [19], so that 11% were in the juvenile age group, 73.4% in the adult group, and 15.6% in the seniors group age. The susceptible population were a total of 3623 individuals [19]; this population was distributed into juvenile, adult, and senior age groups based on the 2015 India's national age distribution [63], adjusted to match the age profile in [19], so that 31.2% were juveniles, 60.6% adults, and 8.3% seniors. The asymptomatic populations were estimated so that 40% of the infected juveniles were asymptomatic [16] and 25% of the infected adults and seniors were asymptomatic, respectively [17, 18].  Table 5 shows the initial conditions used in the simulation of model (32) without age structure.

The low and high effectiveness levels were set to the upper and lower bound, respectively, of the ranges used in the sensitivity analysis and the moderate effectiveness levels were set to the baseline values. It should be pointed out that the parameters values and initial conditions used in these simulations are only of theoretical sense to illustrate the control strategies proposed in this paper.

### 5.1. Mosquito-Reduction Strategy

A reduction in the birth rates (*π*_*M*_) and the average lifespan (*μ*_*M*_) of mosquitoes signifies the effectiveness of adulticiding (such as the use of DDT and indoor residual spraying). For simulation purposes, the following three effectiveness levels of the mosquito-reduction control strategy are considered:

(1) Low effectiveness of the mosquito-reduction strategy: *π*_*M*_ = 500 × 0.32/day, *μ*_*M*_ = (1/21)/day.
(2) Moderate effectiveness of the mosquito-reduction strategy: *π*_*M*_ = 500 × 0.1675/day, *μ*_*M*_ = (1/14)/day.
(3) High effectiveness of the mosquito-reduction strategy: *π*_*M*_ = 500 × 0.015/day, *μ*_*M*_ = (1/7)/day.

**Figure 7:**
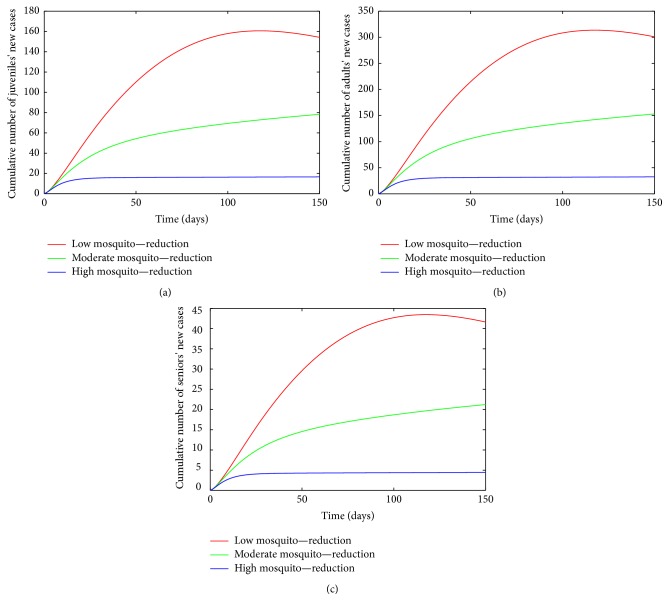
Simulation of age-structured chikungunya model (10) for various effectiveness levels of the mosquito-reduction strategy. (a) The cumulative number of juveniles' new cases. (b) The cumulative number of adults' new cases. (c) The cumulative number of seniors' new cases. Parameter values used are as given in Table 3.

The cumulative number of new cases of infections in juveniles, adults, and seniors (see Figure 7) is simulated for the three effectiveness levels of this strategy. A comparison of the three effectiveness levels in all the three age groups in Table 6 at *t* = 150 days (the end of simulation period) shows that the high-effectiveness mosquito-reduction strategy led to a considerable reduction in the number of new cases; this is followed by the moderate-effectiveness level and the low-effectiveness level produces the most number of new cases. Thus, there is a clear decrease in the cumulative number of new cases with increasing effectiveness level.

A look at Table 6 shows the control profile of model (32) without age structure; as expected, the inclusion of age structure does not change the overall total number of individuals following the implementation of each control strategy. However, the exclusion fails to show the age distribution that will be required for an age based cost effective control strategy.

### 5.2. Personal-Protection Strategy

A reduction in the transmission rates (*β*_*J*_, *β*_*A*_, *β*_*S*_, *β*_*M*_) implies the effectiveness of an individual's protection against mosquito bites, thereby implicitly leading to a reduction in mosquito biting rate (*b*_*M*_). This is typically achieved by using suitable insect repellents or similar products. The following three effectiveness levels of personal protection are considered:

(1) Low effectiveness of the personal-protection strategy: *β*_*J*_ = *β*_*A*_ = *β*_*S*_ = *β*_*M*_ = 0.54/day.
(2) Moderate effectiveness of the personal-protection strategy: *β*_*J*_ = *β*_*A*_ = *β*_*S*_ = *β*_*M*_ = 0.24/day.
(3) High effectiveness of the personal-protection strategy: *β*_*J*_ = *β*_*A*_ = *β*_*S*_ = *β*_*M*_ = 0.15/day.

**Figure 8:**
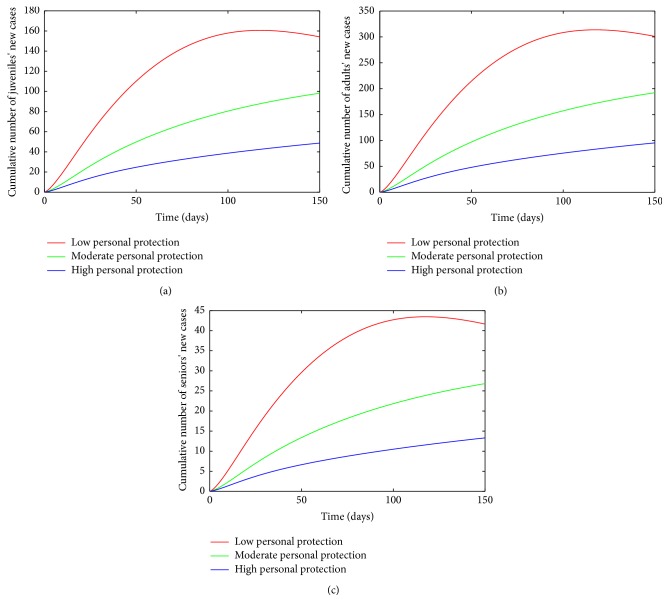
Simulation of age-structured chikungunya model (10) for various effectiveness levels of the personal-protection strategy. (a) The cumulative number of juveniles' new cases. (b) The cumulative number of adults' new cases. (c) The cumulative number of seniors' new cases. Parameter values used are as given in Table 3.

Simulations of the age-structured chikungunya model (10) show a decrease in the cumulative number of new cases with increasing levels of effectiveness (see Figure 8). The high effectiveness personal-protection strategy at *t* = 150 days (the end of simulation period) led to a considerable reduction in the number of new cases compared to the moderate-effectiveness level (see Table 7) at the same time period. The low-effectiveness level performed the poorest producing the most number of new cases.

The control profile of model (32) without age structure and the overall total number of individuals following the implementation of each control strategy is depicted in Table 7 and as expected, the inclusion of age structure does not change the total number of individuals obtained after implementing each control strategy. Table 7 equally shows the flaw in excluding age distribution in the transmission model, as the model lacking age structure fails to show the age distribution required for an efficient and cost effective age based control strategy.

### 5.3. Universal Strategy

Simulations for the universal strategy (where both the mosquito-reduction and personal-protection strategies are implemented at once) are assessed by simulation of the model for the following three effectiveness levels:

(1) Low effectiveness of the universal strategy: *π*_*M*_ = 500 × 0.32/day, *μ*_*M*_ = (1/21)/day, *β*_*J*_ = *β*_*A*_ = *β*_*M*_ = 0.54/day.
(2) Moderate effectiveness of the universal strategy: *π*_*M*_ = 500 × 0.1675/day, *μ*_*M*_ = (1/14)/day, *β*_*J*_ = *β*_*A*_ = *β*_*M*_ = 0.24/day.
(3) High effectiveness of the universal strategy: *π*_*M*_ = 500 × 0.015/day, *μ*_*M*_ = (1/7)/day, *β*_*J*_ = *β*_*A*_ = *β*_*M*_ = 0.15/day.

**Figure 9:**
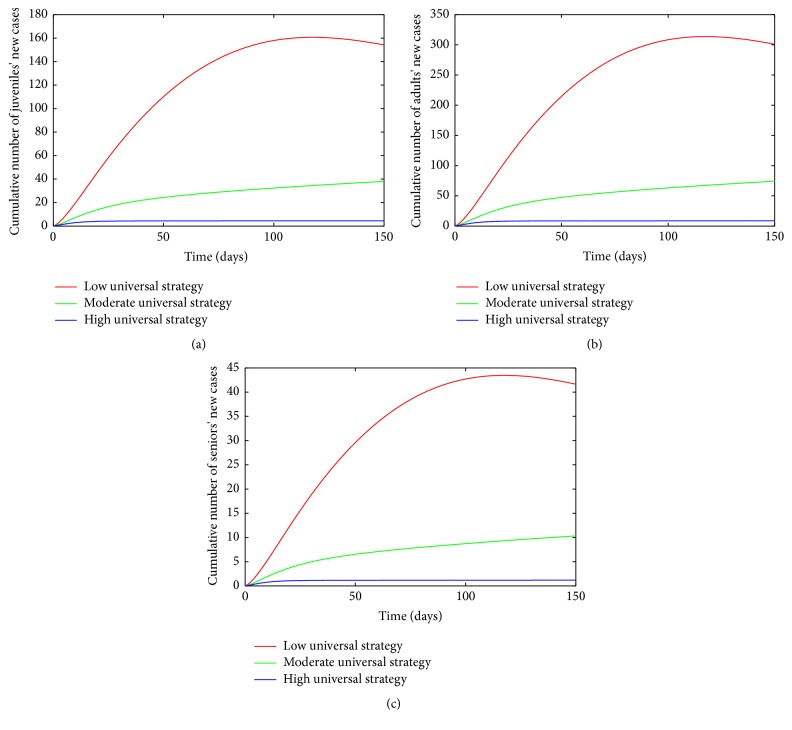
Simulation of age-structured chikungunya model (10) for various effectiveness levels of the universal strategy. (a) The cumulative number of juveniles' new cases. (b) The cumulative number of adults' new cases. (c) The cumulative number of seniors' new cases. Parameter values used are as given in Table 3.

The cumulative number of new cases of infections in juveniles, adults, and seniors is simulated for the three levels of effectiveness for the universal strategy (see Figure 9). A comparison of the three effectiveness levels in Table 8 at *t* = 150 days shows that the high-effectiveness level leads to a considerable reduction in the number of new cases; this is followed by the moderate-effectiveness level and the low-effectiveness level produced the most number of new cases.

A comparison of the various high-effectiveness levels of the three control strategies (mosquito-reduction, personal-protection, and universal strategies) in each individual age group at *t* = 150 days (see Table 9) shows as expected that the universal strategy is more effective than the other two strategies implemented separately. This is followed by mosquito-reduction strategy which is more effective than the personal protection strategy in reducing chikungunya disease burden.

Table 9 also shows the control profile of model (32) without age structure; as expected, the inclusion of age structure does not change the overall total number of individuals following the implementation of each control strategy. However, the exclusion fails to show the detailed age distribution that will be required for an effective age based control strategy which in turn will be cost effective.

## 6. Discussion and Conclusion

In this paper, a new deterministic model is designed and used to study the transmission dynamics of an age-structured chikungunya model. The model stratified the population by age into juveniles, adults, and seniors and incorporates notable features such as the inclusion of asymptomatic and symptomatic individuals. In order to reduce the number of chikungunya cases, three different control strategies (involving mosquito-reduction strategy, personal-protection strategy, and universal strategy) with three different effectiveness levels (low, moderate, and high) were implemented.

The study shows that the disease-free equilibrium of the model is locally and globally asymptotically stable whenever the associated reproduction number (*ℛ*_0_, an epidemiological threshold quantity that measures the spreading capacity of the disease) is less than unity and unstable otherwise. The model is shown from this study to exhibit in the presence of disease induced mortality the phenomenon of backward bifurcation, where the stable disease-free equilibrium coexists with a stable endemic equilibrium, when the associated reproduction number is less than unity. Furthermore, the study shows that the inclusion of age structure to the chikungunya virus transmission model does not alter its qualitative dynamics with respect to the local and global stability of the disease-free equilibrium (DFE), as well as with respect to its backward bifurcation property.

This study identifies (via sensitivity analysis) the dominant parameters using as model outcome the basic reproduction number. The parameters with the largest impact are the mosquito biting rate, the transmission probability* per* contact in mosquitoes and in humans, mosquito recruitment rate, and the death rate of the mosquitoes. The study further shows that the inclusion of age structure does not alter the sensitivity and dominance of the dominant parameters of the chikungunya virus transmission model. The identification of these key parameters is vital to the formulation of effective control strategies for combating the spread of the disease. In other words, the results of this sensitivity analysis suggest that a strategy that reduces the mosquito biting rate, the mosquito recruitment rate, and the transmission probability per contact in mosquitoes and in humans and increases the death rate of the mosquito will be effective in curtailing the spread of chikungunya virus in the community.

Thus, this study shows that even though age distribution is observed in the various epidemics in India [19, 20], Thailand [21], and Reunion Islands [5, 22] and across Europe [23], the inclusion of age does not alter the qualitative dynamics of the chikungunya virus transmission model. This implies that the transmission dynamics can be adequately studied without including age structure as observed in the epidemic areas. However, the exclusion of the age structure fails to show the age distribution necessary for adequate and effective control, as shown in Tables 6, 7, 8, and 9. In other words, the model with the exclusion of age structure will lead to a one size fits all blanket control for the entire population.

In order to reduce the number of infected cases, different parameters values were adjusted using the results from the sensitivity analysis. The control strategies were implemented for several cases (mosquito-reduction strategy, personal-protection strategy, and universal strategy) with three different effectiveness levels (low, moderate, and high) using as output measure the cumulative number of new cases of infections in juveniles, adults, and seniors. The results show that the cumulative number of new cases of infections in juveniles, adults, and seniors decreases with increasing effectiveness level, with the high-effectiveness level producing a considerable reduction in the number of new cases. Further comparison of the three control strategies (mosquito reduction, personal-protection, and universal strategies) shows that the universal strategy is more effective in reducing the number of new cases than the other two strategies implemented separately. This is followed by mosquito-reduction strategy which is more effective than the personal-protection strategy in reducing chikungunya disease burden in the community. However, to determine the best and most cost effective strategy, a cost-effectiveness analysis will need to be carried out [64]; this is a future work that is been considered in another paper.

Hence, in this paper, we formulated and analyzed a system of ordinary differential equations for an age-structure transmission dynamics of chikungunya virus. Some of theoretical and epidemiological findings of this study are summarized below:

(i) The age-structured chikungunya model (10) is locally and globally asymptotically stable (LAS) when *ℛ*_0_ < 1 and unstable when *ℛ*_0_ > 1.
(ii) The model exhibits in the presence of disease induced mortality the phenomenon of backward bifurcation, where the stable disease-free equilibrium coexists with a stable endemic equilibrium, when the associated reproduction number is less than unity.
(iii) The inclusion of age structure to the chikungunya transmission model (10) does not alter its qualitative dynamics with respect to the local and global stability of the DFE, as well as with respect to its backward bifurcation property.
(iv) The sensitivity analysis of the model shows that the dominant parameters are the mosquito biting rate (*b*_*M*_), the transmission probability* per* contact in mosquitoes (*β*_*M*_) and in humans (*β*_*S*_), mosquito recruitment rate (*π*_*M*_), and the death rate of the mosquitoes (*μ*_*M*_).
(v) The inclusion of age structure does not alter the sensitivity and dominance of the dominant parameters of the age-structured chikungunya model (10).
(vi) The numerical simulations reveal that the exclusion of age structure fails to show the age distribution needed for an age based effective control strategy, leading to a one size fits all blanket control for the entire population.
(vii) Numerical simulations indicate that mosquito-reduction strategy is more effective than personal-protection strategy, while the universal strategy is the most effective strategy in reducing chikungunya disease burden in the community.

## Figures and Tables

**Table 1:** Description of the variables and parameters of the age-structured chikungunya model (10).

Variable	Description
*S*_*J*_, *S*_*A*_, *S*_*S*_	Population of susceptible juvenile, adult, and senior humans
*E*_*J*_, *E*_*A*_, *E*_*S*_	Population of exposed juvenile, adult, and senior humans
*I*_*AJ*_, *I*_*SJ*_	Population of asymptomatic and symptomatic juvenile humans
*I*_*AA*_, *I*_*SA*_	Population of asymptomatic and symptomatic adult humans
*I*_*AS*_, *I*_*SS*_	Population of asymptomatic and symptomatic senior humans
*R*_*J*_, *R*_*A*_, *R*_*S*_	Population of recovered juvenile, adult, and senior humans
*S*_*M*_	Population of susceptible mosquitoes
*E*_*M*_	Population of exposed mosquitoes
*I*_*M*_	Population of infectious mosquitoes

Parameter	Description

*π*_*J*_	Recruitment rate of juvenile humans
*π*_*M*_	Recruitment rate of mosquitoes
*α*, *ξ*	Juvenile and adult maturation rates
*β*_*J*_, *β*_*A*_, *β*_*S*_	Transmission probability per contact for susceptible humans
*β*_*M*_	Transmission probability per contact for susceptible mosquitoes
*b*_*M*_	Mosquito biting rate
*μ*_*J*_, *μ*_*A*_, *μ*_*S*_	Natural death rate of juvenile, adult, and senior humans
*μ*_*M*_	Natural death rate of mosquitoes
*ε*_*J*_, *ε*_*A*_, *ε*_*S*_	Fraction of exposed humans becoming asymptomatic and symptomatic
*σ*_*J*_, *σ*_*A*_, *σ*_*S*_	Progression rate of exposed juvenile, adult, and senior humans
*γ*_*AJ*_, *γ*_*SJ*_	Recovery rate of asymptomatic and symptomatic juvenile humans
*γ*_*AA*_, *γ*_*SA*_	Recovery rate of asymptomatic and symptomatic adult humans
*γ*_*AS*_, *γ*_*SS*_	Recovery rate of asymptomatic and symptomatic senior humans
*σ*_*M*_	Progression rate of exposed mosquitoes

**Table 2:** Number of possible positive real roots of *f*(*x*) for *ℛ*_0_ > 1.

Cases	*c*_3_	*c*_2_	*c*_1_	*c*_0_	*ℛ*_0_	Number of sign changes	Number of possible positive real roots (endemic equilibrium)
1	+	+	+	−	*ℛ*_0_ > 1	1	1
2	+	−	−	−	*ℛ*_0_ > 1	1	1
3	+	+	−	−	*ℛ*_0_ > 1	1	1

**Table 3:** Parameters values of models (10) and (32).

Parameter	Values	Range	References
*π*_*J*_, *π*_*H*_	$400\times \frac{1}{15\times 365}$	$400\times \frac{1}{15\times 365}\text{–}400\times \frac{1}{12\times 365}$	[26, 31]
*α*	$\frac{1}{16\times 365}$	$\frac{1}{18\times 365}\text{–}\frac{1}{15\times 365}$	Assumed
*ξ*	$\frac{1}{50\times 365}$	$\frac{1}{55\times 365}\text{–}\frac{1}{45\times 365}$	Estimated
*β*_*J*_, *β*_*A*_, *β*_*S*_, *β*_*H*_	0.24	0.001–0.54	[25, 26, 29, 32, 33]
*b*_*M*_	0.25	0.19–0.39	[26, 34]
*μ*_*J*_	$\frac{1}{3\times 365}$	$\frac{1}{5\times 365}\text{–}\frac{1}{1\times 365}$	Assumed
*μ*_*A*_	$\frac{1}{40\times 365}$	$\frac{1}{60\times 365}\text{–}\frac{1}{18\times 365}$	Assumed
*μ*_*S*_	$\frac{1}{70\times 365}$	$\frac{1}{80\times 365}\text{–}\frac{1}{60\times 365}$	Assumed
*μ*_*H*_	$\frac{1}{70\times 365}$	$\frac{1}{76\times 365}\text{–}\frac{1}{68\times 365}$	[26, 31]
*ε*_*J*_, *ε*_*A*_, *ε*_*S*_, *ε*_*H*_	0.155	0.03–0.28	[17]
*σ*_*J*_, *σ*_*S*_	$\frac{1}{2\times 3}$	$\frac{1}{2\times 4}\text{–}\frac{1}{2\times 2}$	Assumed
*σ*_*A*_, *σ*_*H*_	$\frac{1}{3}$	$\frac{1}{4}\text{–}\frac{1}{2}$	[5, 26, 32, 35–37]
*γ*_*AJ*_, *γ*_*SJ*_	$\frac{1}{1.5\times 4.5}$	$\frac{1}{1.5\times 8}\text{–}\frac{1}{1.5\times 3}$	Assumed
*γ*_*AA*_, *γ*_*SA*_	$\frac{1}{4.5}$	$\frac{1}{7}\text{–}\frac{1}{3}$	[26, 28, 36, 37]
*γ*_*AS*_, *γ*_*SS*_	$\frac{1}{2.5\times 4.5}$	$\frac{1}{2.5\times 8}\text{–}\frac{1}{2.5\times 3}$	Assumed
*γ*_*AH*_, *γ*_*SH*_	$\frac{1}{4.5}$	$\frac{1}{7}\text{–}\frac{1}{3}$	[26, 28, 36, 37]
*π*_*M*_	500 × 0.1675	500 × 0.015–500 × 0.32	[26, 31, 38, 39]
*β*_*M*_	0.24	0.005–0.35	[25, 29, 33, 40, 41]
*σ*_*M*_	$\frac{1}{3.5}$	$\frac{1}{6}\text{–}\frac{1}{2}$	[6, 28, 32, 36]
*μ*_*M*_	$\frac{1}{14}$	$\frac{1}{42}\text{–}\frac{1}{14}$	[28, 32, 35–37]

**Table 4:** Initial conditions used in the simulations of model (10) with age structure.

*S*_*J*_(0) = 1130	*E*_*J*_(0) = 123	*I*_*AJ*_(0) = 84	*I*_*SJ*_(0) = 211	*R*_*J*_(0) = 0
*S*_*A*_(0) = 2196	*E*_*A*_(0) = 264	*I*_*AA*_(0) = 351	*I*_*SA*_(0) = 1404	*R*_*A*_(0) = 0
*S*_*S*_(0) = 300	*E*_*S*_(0) = 224	*I*_*AS*_(0) = 75	*I*_*SS*_(0) = 298	*R*_*S*_(0) = 0
*S*_*M*_(0) = 500	*E*_*M*_(0) = 100	*I*_*M*_(0) = 250.		

**Table 5:** Initial conditions used in the simulations of model (32) without age structure.

*S*_*H*_(0) = 3623	*E*_*H*_(0) = 611	*I*_*AH*_(0) = 510	*I*_*SH*_(0) = 1913	*R*_*H*_(0) = 0
*S*_*M*_(0) = 500	*E*_*M*_(0) = 100	*I*_*M*_(0) = 250.		

**Table 6:** Simulation results of the cumulative number of new cases at *t* = 150 days for the age-structured chikungunya model (10) using mosquito-reduction strategy and the control profile of model (32) without age structure.

Humans	Low effectiveness	Moderate effectiveness	High effectiveness
Juveniles	154.1849	78.3505	16.6460
Adults	300.8889	153.0260	32.3984
Seniors	41.6331	21.2259	4.4462
Without age structure	471.2875	184.6136	51.9926

**Table 7:** Simulation results of the cumulative number of new cases at *t* = 150 days for the age-structured chikungunya model (10) using personal-protection strategy and the control profile of model (32) without age structure.

Humans	Low effectiveness	Moderate effectiveness	High effectiveness
Juveniles	154.1849	98.2295	48.7211
Adults	300.8889	192.2149	95.3561
Seniors	41.6331	26.8144	13.3071
Without age structure	471.2875	192.5576	91.4392

**Table 8:** Simulation results of the cumulative number of new cases at *t* = 150 days for the age-structured chikungunya model (10) using universal strategy and the control profile of model (32) without age structure.

Humans	Low effectiveness	Moderate effectiveness	High effectiveness
Juveniles	154.1849	38.0114	4.4823
Adults	300.8889	74.2880	8.7228
Seniors	41.6331	10.3224	1.1966
Without age structure	471.2875	81.0076	14.1767

**Table 9:** Comparison of the cumulative number of new cases at *t* = 150 days for the high-effectiveness levels of the three control strategies for the age-structured chikungunya model (10) and the control profile of model (32) without age structure.

Human Age groups	Mosquito-reduction strategy	Personal-protection strategy	Universal strategy
Juveniles	16.6460	48.7211	4.4823
Adults	32.3984	95.3561	8.7228
Seniors	4.4462	13.3071	1.1966
Without age structure	51.9926	91.4392	14.1767

## Appendices

### A. Derivation of (1)

Humans (susceptible and infected) are bitten by mosquitoes; thus, the average number of mosquito bites humans receive (denoted by *b*_*M*_) depends on the humans in the community and the total size of the populations of mosquitoes [65]. Thus, it is reasonable to assume that the biting rate *b*_*M*_ is constant as female mosquitoes have certain number of blood meals over their lifetime [66]. Let *b*_*H*_ be the rate at which bites are received by a single host per unit time. Thus, for the number of bites to be conserved, the following conservation law must hold (i.e., the total number of bites by mosquitoes equals the total number of bites received by humans):

(A.1)
$$\begin{array}{c} b_{M}N_{M}=b_{H}\left(\;N_{H},N_{M}\;\right)N_{H}, \end{array}$$

so that

(A.2)
$$\begin{array}{c} N_{M}=\frac{b_{H}\left(N_{H},N_{M}\right)N_{H}}{b_{M}}. \end{array}$$

Let *β*_*J*_ *b*_*H*_ be the effective contact rate between a susceptible juvenile and infectious mosquitoes, where *β*_*J*_ is the transmission probability per contact from an infectious mosquito to a susceptible juvenile. Similarly, let *β*_*M*_ *b*_*M*_ be the effective contact rate between a susceptible mosquito and infectious juveniles, where *β*_*M*_ is the transmission probability per contact from an infectious juvenile to a susceptible mosquito. Thus, susceptible juveniles acquire infection, following effective contact with an infectious vector, at a rate *λ*_*J*_, given by

(A.3)
$$\begin{array}{c} \lambda_{J}=\frac{\beta_{J}b_{H}\left(N_{H},N_{M}\right)I_{M}}{N_{M}}. \end{array}$$

Using (A.1) in (A.3) gives

(A.4)
$$\begin{array}{c} \lambda_{J}=\frac{\beta_{J}b_{M}I_{M}}{N_{H}}. \end{array}$$

### B. Proof of Lemma 1

Proof Let *t*_1_ = sup{*t* > 0 : *F*(*t*) > 0 ∈ [0, *t*]}. Thus, *t*_1_ > 0. It follows from the first equation of system (10) that

(B.1)
$$\begin{array}{c} \frac{dS_{J}}{dt}=\pi_{J}-\frac{\beta_{J}b_{M}S_{J}I_{M}}{N_{H}}-\alpha S_{J}-\mu_{J}S_{J}, \end{array}$$

which can be rewritten as

(B.2)
ddtSJtexp⁡∫0t1βJbMIMζNHζdζ+k1t=πJexp⁡∫0t1βJbMIMζNHζdζ+k1t,

where *k*_1_ = *α* + *μ*_*J*_. Hence,

(B.3)
SJt1exp⁡∫0t1βJbMIMζNHζdζ+k1t1−SJ0=∫0t1πJexp⁡∫0pβJbMIMζNHζdζ+k1pdp

so that

(B.4)
SJt1=SJ0exp⁡−∫0t1βJbMIMζNHζdζ+k1t1+exp⁡−∫0t1βJbMIMζNHζdζ+k1t1·∫0t1πJexp⁡∫0pβJbMIMζNHζdζ+k1pdp>0.

Similarly, it can be shown that *F* > 0 for all *t* > 0. For the second part of the proof, note that 0 < *E*_*J*_(*t*) ≤ *N*_*H*_(*t*), 0 < *I*_*SJ*_(*t*) ≤ *N*_*H*_(*t*), 0 < *I*_*AJ*_(*t*) ≤ *N*_*H*_(*t*), 0 < *R*_*J*_(*t*) ≤ *N*_*H*_(*t*), 0 < *S*_*A*_(*t*) ≤ *N*_*H*_(*t*), 0 < *E*_*A*_(*t*) ≤ *N*_*H*_(*t*), 0 < *I*_*AA*_(*t*) ≤ *N*_*H*_(*t*), 0 < *I*_*SA*_(*t*) ≤ *N*_*H*_(*t*), 0 < *R*_*A*_(*t*) < *N*_*H*_(*t*), 0 < *S*_*S*_(*t*) ≤ *N*_*H*_(*t*), 0 < *E*_*S*_(*t*) ≤ *N*_*H*_(*t*), 0 < *I*_*AS*_(*t*) ≤ *N*_*H*_(*t*), 0 < *I*_*SS*_(*t*) ≤ *N*_*H*_(*t*), 0 < *R*_*S*_(*t*) ≤ *N*_*H*_(*t*), 0 < *S*_*M*_(*t*) ≤ *N*_*M*_(*t*), 0 < *E*_*M*_(*t*) ≤ *N*_*M*_(*t*), 0 < *I*_*M*_(*t*) ≤ *N*_*M*_(*t*). Adding the human and mosquito component of the age-structured chikungunya model (10) gives

(B.5)
dNHtdt=πJ−μJNHt,dNMtdt=πM−μMNMt,

where *μ*_*H*_ = min⁡{*μ*_*J*_, *μ*_*A*_, *μ*_*S*_}. Hence,

(B.6)
πJμH≤liminft→∞⁡ NHt≤limsupt→∞⁡ NHt=πJμH,πMμM≤liminft→∞⁡ NMt=limsupt→∞⁡ NMt=πMμM

as required.

### C. Proof of Lemma 2

Proof The following steps are followed to establish the positive invariance of *Ω* (i.e., solutions in *Ω* remain in *Ω* for all *t* > 0). The rate of change of the total population is obtained by adding the human and mosquito component of the age-structured chikungunya model (10) to give

(C.1)
dNHtdt=πJ−μHNHt,dNMtdt=πM−μMNMt,

where *μ*_*H*_ = min⁡{*μ*_*J*_, *μ*_*A*_, *μ*_*S*_}. A standard comparison theorem [67] can then be used to show that *N*_*H*_(*t*) ≤ *N*_*H*_(0)*e* ^−*μ*_*H*_ *t*^ + (*π*_*J*_/*μ*_*H*_)(1 − *e* ^−*μ*_*H*_ *t*^) and *N*_*M*_(*t*) ≤ *N*_*M*_(0)*e* ^−*μ*_*M*_ *t*^ + (*π*_*M*_/*μ*_*M*_)(1 − *e* ^−*μ*_*M*_ *t*^). In particular, *N*_*H*_(*t*) ≤ *π*_*J*_/*μ*_*H*_, if *N*_*H*_(0) ≤ *π*_*J*_/*μ*_*H*_ and *N*_*M*_(*t*) ≤ *π*_*M*_/*μ*_*M*_, if *N*_*M*_(0) ≤ *π*_*M*_/*μ*_*M*_. Thus, the region *Ω* is positively invariant. Hence, it is sufficient to consider the dynamics of the flow generated by (10) in *Ω*. In this region, the model is epidemiologically and mathematically well posed [45]. Thus, every solution of the age-structured chikungunya model (10) with initial conditions in *Ω* remains in *Ω* for all *t* > 0. Therefore, the *ω*-limit sets of the system (10) are contained in *Ω*. This result is summarized below.

### D. Proof of Lemma 4

Proof It follows from the first equation of age-structured chikungunya model (10) (where *S*_*J*_^*∗*^ = *π*_*J*_/(*α* + *μ*_*J*_)) that

(D.1)
dSJtdtπJ−βJbMSJIMNH−α+μJSJt≤πJ−α+μJSJt≤α+μJπJα+μH−SJt=α+μJSJ∗−SJt.

Hence,

(D.2)
$$\begin{array}{c} S_{J}\left(\;t\;\right)\leq S_{J}^{*}-\left[\;S_{J}^{*}-S_{J}\left(\;0\;\right)\;\right]e^{-\left(\alpha +\mu_{J}\right)t}. \end{array}$$

Thus, if *N*_*H*_^*∗*^ = Π_*J*_/*μ*_*H*_ and *S*_*J*_(0) ≤ *S*_*J*_^*∗*^ for all *t* ≥ 0, then *S*_*J*_(*t*) ≤ *S*_*J*_^*∗*^ for all *t* ≥ 0. Similarly, it follows from the sixth equation of age-structured chikungunya model (10) (where *S*_*A*_^*∗*^ = (*π*_*J*_/(*α* + *μ*_*J*_))(*α*/(*ξ* + *μ*_*A*_)) that

(D.3)
dSAtdtαSJt−βAbMSAIMNH−ξ+μASAt≤αSJt−ξ+μASAt≤αSJ∗−ξ+μASAt,since  SJ<SJ∗απJα+μJ−ξ+μASAt≤ξ+μHπJα+μJαξ+μA−SAt=ξ+μASA∗−SAt.

Thus,

(D.4)
$$\begin{array}{c} S_{A}\left(\;t\;\right)\leq S_{A}^{*}-\left[\;S_{A}^{*}-S_{A}\left(\;0\;\right)\;\right]e^{-\left(\xi +\mu_{A}\right)t}. \end{array}$$

Thus, if *S*_*A*_(0) ≤ *S*_*A*_^*∗*^ for all *t* ≥ 0, then *S*_*A*_(*t*) ≤ *S*_*A*_^*∗*^ for all *t* ≥ 0. Furthermore, it follows from the eleventh equation of age-structured chikungunya model (10) (where *S*_*S*_^*∗*^ = *ξπ*_*J*_ *α*/*μ*_*S*_(*α* + *μ*_*J*_)(*ξ* + *μ*_*A*_)) that

(D.5)
dSStdtξSAt−βSbMSSIMNH−μSSSt≤ξSAt−μSSSt≤ξSA∗t−μSSSt,since  SA<SA∗ξπJαα+μJξ+μA−μSSSt≤μSξπJαμSα+μJξ+μA−SSt=μSSS∗−SSt.

Thus,

(D.6)
$$\begin{array}{c} S_{S}\left(\;t\;\right)\leq S_{S}^{*}-\left[\;S_{S}^{*}-S_{S}\left(\;0\;\right)\;\right]e^{-\mu_{S}t}. \end{array}$$

Hence, if *S*_*S*_(0) ≤ *S*_*S*_^*∗*^ for all *t* ≥ 0, then *S*_*S*_(*t*) ≤ *S*_*S*_^*∗*^ for all *t* ≥ 0. Finally, it follows from the fifteenth equation of age-structured chikungunya model (10) that

(D.7)
dSMtdt=πM−βMbMIAJ+ISJ+IAA+ISA+IAS+ISSNHSM−μMSMt≤πM−μMSMt≤μMπMμM−SMt=μMSM∗−SMt.

Thus,

(D.8)
$$\begin{array}{c} S_{M}\left(\;t\;\right)\leq S_{M}^{*}-\left[\;S_{M}^{*}-S_{M}\left(\;0\;\right)\;\right]e^{-\mu_{M}t}. \end{array}$$

Hence, if *N*_*M*_^*∗*^ = *π*_*M*_/*μ*_*M*_ and *S*_*M*_(0) ≤ *S*_*M*_^*∗*^ for all *t* ≥ 0, then *S*_*M*_(*t*) ≤ *S*_*M*_^*∗*^ for all *t* ≥ 0. Thus, in summary, it has been shown that the region *Ω*_1_ is positively invariant and attracts all solutions in *ℝ*_+_^18^ for the age-structured model (10).

### E. Proof of Theorem 5

Proof To prove the global stability of the DFE, we will follow the approach in [68]. Let *X* = (*S*_*J*_, *R*_*J*_, *S*_*A*_, *R*_*A*_, *S*_*S*_, *R*_*S*_, *S*_*M*_) and *Z* = (*E*_*J*_, *I*_*AJ*_, *I*_*SJ*_, *E*_*A*_, *I*_*AA*_, *I*_*SA*_, *E*_*S*_, *I*_*AS*_, *I*_*SS*_, *E*_*M*_, *I*_*M*_) and group system (10) into

(E.1)
dXdt=FX,0,dZdt=GX,Z,

where *F*(*X*, 0) is the right hand side of ${\dot{S_{J}}}_{}$ , ${\dot{R_{J}}}_{}$ , ${\dot{S_{A}}}_{}$ , ${\dot{R_{A}}}_{}$ , ${\dot{S_{S}}}_{}$ , ${\dot{R_{S}}}_{}$ , and ${\dot{S_{M}}}_{}$ with *E*_*J*_ = *I*_*AJ*_ = *I*_*SJ*_ = *E*_*A*_ = *I*_*AA*_ = *I*_*SA*_ = *E*_*S*_ = *I*_*AS*_ = *I*_*SS*_ = *E*_*M*_ = *I*_*M*_ = 0 and *G*(*X*, *Z*) is the right hand side of ${\dot{E_{J}}}_{}$ , ${\dot{I_{AJ}}}_{}$ , ${\dot{I_{SJ}}}_{}$ , ${\dot{E_{A}}}_{}$ , ${\dot{I_{AA}}}_{}$ , ${\dot{I_{SA}}}_{}$ , ${\dot{E_{S}}}_{}$ , ${\dot{I_{AS}}}_{}$ , ${\dot{I_{SS}}}_{}$ , ${\dot{E_{M}}}_{}$ , and ${\dot{I_{M}}}_{}.$

Next, consider the reduced system *dX*/*dt* = *F*(*X*, 0) given as follows:

(E.2)
dSJdt=πJ−α+μJSJ,dRJdt=−α+μJRJ,dSAdt=αSJ−ξ+μASA,dRAdt=αRJ−ξ+μARA,

(E.3)
dSSdt=ξSA−μSSS,dRSdt=ξRA−μSRS,dSMdt=πM−μMSM.

Let

(E.4)
X∗SJ∗,RJ∗,SA∗,RA∗,SS∗,RS∗,SM∗=πJα+μJ,0,αSJ∗ξ+μA,0,ξSA∗μS,0,πMμM

be an equilibrium of the reduced system (E.2) and (E.3); we show that *X* ^*∗*^ is a globally stable equilibrium in *Ω*_1_. To do this, solve the first and second equation of (E.2) and (E.3); this gives *S*_*J*_(*t*) = *π*_*J*_/*l*_1_ + *e* ^−*l*_1_ *t*^[*S*_*J*_(0) − *π*_*J*_/*l*_1_], *R*_*J*_(*t*) = *R*_*J*_(0)*e* ^−*l*_1_ *t*^ (where *l*_1_ = *α* + *μ*_*J*_), which approaches *π*_*J*_/*l*_1_ and zero, respectively, as *t* → *∞*. Next, solving for *S*_*A*_(*t*) and *R*_*A*_(*t*) and using *S*_*J*_(*t*) and *R*_*J*_(*t*) in (E.2) and (E.3) give *S*_*A*_(*t*) = [∫_0_^*t*^ *αS*_*J*_(*z*)*e* ^*l*_2_ *z*^ *dz* + *S*_*A*_(0)]*e* ^−*l*_2_ *t*^ and *R*_*A*_(*t*) = [∫_0_^*t*^ *αR*_*J*_(*z*)*e* ^*l*_2_ *z*^ *dz* + *R*_*A*_(0)]*e* ^−*l*_2_ *t*^ (where *l*_2_ = *ξ* + *μ*_*A*_), which converges, respectively, to *π*_*J*_ *α*/*l*_1_ *l*_2_ and zero as *t* → *∞*. Similarly, solving for *S*_*S*_(*t*) and *R*_*S*_(*t*) and using *S*_*A*_(*t*) and *R*_*A*_(*t*) in (E.2) give *S*_*S*_(*t*) = [∫_0_^*t*^ *ξS*_*A*_(*z*)*e* ^*μ*_*S*_ *z*^ *dz* + *S*_*S*_(0)]*e* ^−*μ*_*S*_ *t*^ and *R*_*S*_(*t*) = [∫_0_^*t*^ *αR*_*A*_(*z*)*e* ^*μ*_*S*_ *z*^ *dz* + *R*_*S*_(0)]*e* ^−*μ*_*S*_ *t*^ which converges to *π*_*J*_ *αξ*/*l*_1_ *l*_2_ *μ*_*S*_ and zero as *t* → *∞*. Lastly solving for *S*_*M*_(*t*) in (E.2) gives *S*_*M*_(*t*) = *π*_*M*_/*μ*_*M*_ + *e* ^−*μ*_*M*_ *t*^[*S*_*M*_(0) − *π*_*M*_/*μ*_*M*_] which converges to *π*_*M*_/*μ*_*M*_, as *t* → *∞*. These asymptotic dynamics are independent of initial conditions in *Ω*. Hence, the convergence of solutions of (E.2) and (E.3) is global in *Ω*_1_. Next, we require *G*(*X*, *Z*) to satisfy the following two conditions given in [69, page 246], namely,

(i) *G*(*X*, 0) = 0;
(ii) $G(X,Z)=D_{Z}G(X^{*},0)Z-\hat{G}(X,Z)$ , $\hat{G}(X,Z)\geq 0$ ,

where (*X* ^*∗*^, 0) = (*π*_*J*_/(*α* + *μ*_*J*_), 0, *αS*_*J*_^*∗*^/(*ξ* + *μ*_*A*_), 0, *ξS*_*A*_^*∗*^/*μ*_*S*_, 0, *π*_*M*_/*μ*_*M*_, 0,0, 0,0, 0,0, 0,0, 0,0, 0) and *D*_*Z*_ *G*(*X* ^*∗*^, 0) is the Jacobian of *G*(*X*, *Z*) taken with respect to (*E*_*J*_, *I*_*AJ*_, *I*_*SJ*_, *E*_*A*_, *I*_*AA*_, *I*_*SA*_, *E*_*S*_, *I*_*AS*_, *I*_*SS*_, *E*_*M*_, *I*_*M*_) and evaluated at (*X* ^*∗*^, 0), which is an *M*-matrix (the off diagonal elements are nonnegative). Thus,

(E.5)

where Ψ_*J*_ = *β*_*J*_ *b*_*M*_ *S*_*J*_^*∗*^/*N*_*H*_^*∗*^, Ψ_*A*_ = *β*_*A*_ *b*_*M*_ *S*_*A*_^*∗*^/*N*_*H*_^*∗*^, Ψ_*S*_ = *β*_*S*_ *b*_*M*_ *S*_*S*_^*∗*^/*N*_*H*_^*∗*^, Ψ_*M*_ = *b*_*M*_ *β*_*M*_ *S*_*M*_^*∗*^/*N*_*H*_^*∗*^, and

(E.6)

where Φ_*J*_ = *β*_*J*_ *b*_*M*_(*S*_*J*_^*∗*^/*N*_*H*_^*∗*^)((1 − (*N*_*H*_^*∗*^/*S*_*J*_^*∗*^)(*S*_*J*_/*N*_*H*_)), Φ_*A*_ = *β*_*A*_ *b*_*M*_(*S*_*A*_^*∗*^/*N*_*H*_^*∗*^)(1 − (*N*_*H*_^*∗*^/*S*_*A*_^*∗*^)(*S*_*A*_/*N*_*H*_)), Φ_*S*_ = *β*_*S*_ *b*_*M*_(*S*_*S*_^*∗*^/*N*_*H*_^*∗*^)(1 − (*N*_*H*_^*∗*^/*S*_*S*_^*∗*^)(*S*_*S*_/*N*_*H*_)), and Φ_*M*_ = *b*_*M*_ *β*_*M*_(*S*_*M*_^*∗*^/*N*_*H*_^*∗*^)(1 − (*N*_*H*_^*∗*^ *S*_*M*_/*S*_*M*_^*∗*^ *N*_*H*_)). Furthermore, *S*_*J*_^*∗*^ = (*π*_*J*_/*k*_1_), *S*_*A*_^*∗*^ = (*π*_*J*_ *α*/*k*_1_ *k*_6_), *S*_*S*_^*∗*^ = (*π*_*J*_ *αξ*/*k*_1_ *k*_6_ *μ*_*H*_), *N*_*H*_^*∗*^ = (*π*_*H*_/*μ*_*H*_), and *S*_*M*_^*∗*^ = (*π*_*M*_/*μ*_*M*_). We have in *Ω*_1_ that, *S*_*J*_ ≤ *S*_*J*_^*∗*^, *S*_*A*_ ≤ *S*_*A*_^*∗*^, *S*_*S*_ ≤ *S*_*S*_^*∗*^, and also *S*_*M*_ ≤ *S*_*M*_^*∗*^. Thus, if the human population is at equilibrium level, it follows that (1 − (*N*_*H*_^*∗*^/*S*_*J*_^*∗*^)(*S*_*J*_/*N*_*H*_)) > 0, (1 − (*N*_*H*_^*∗*^/*S*_*A*_^*∗*^)(*S*_*A*_/*N*_*H*_)) > 0, (1 − (*N*_*H*_^*∗*^/*S*_*S*_^*∗*^)(*S*_*S*_/*N*_*H*_)) > 0, and (1 − (*S*_*M*_ *N*_*H*_^*∗*^/*N*_*H*_ *S*_*M*_^*∗*^)) > 0; hence ${\hat{G}}_{2}\left(X,Z\right)\geq 0$ . Therefore, by the theorem in [69, page 246], the disease-free equilibrium is globally asymptotically stable since in the absence of disease induced mortality the human population is constant.

### F. Components of the Equilibrium *ℰ*_1_ of Model (29)

Consider

(F.1)
SJ∗∗=πJ2βJbMIM∗∗μH+k1πJ,EJ∗∗=βJbMIM∗∗μHπJk2βJbMIM∗∗μH+k1πJ,IAJ∗∗=εJσJβJbMIM∗∗μHπJk3k2βJbMIM∗∗μH+k1πJ,ISJ∗∗=1−εJσJβJbMIM∗∗μHπJk4k2βJbMIM∗∗μH+k1πJ,RJ∗∗=σJβJbMIM∗∗μHπJγAJk4εJ+γSJ1−εJk3k5k4k3k2βJbMIM∗∗μH+k1πJ,SA∗∗=αSJ∗∗πJβAbMIM∗∗μH+πJk6,EA∗∗=αEJ∗∗βAbMIM∗∗μH+EJ∗∗πJk6+βAbMIM∗∗μHSJ∗∗k7βAbMIM∗∗μH+πJk6,IAA∗∗=αIAJ∗∗k7βAbMIM∗∗μH+IAJ∗∗k7πJk6+εAσAEJ∗∗βAbMIM∗∗μH+εAσAEJ∗∗πJk6+εAσAβAbMIM∗∗μHSJ∗∗k8k7βAbMIM∗∗μH+πJk6,ISA∗∗=αISJ∗∗k7βAbMIM∗∗μH+ISJ∗∗k7πJk6+1−εJσAEJ∗∗βAbMIM∗∗μH+1−εJσAEJ∗∗πJk6+1−εJσAβAbMIM∗∗μHSJ∗∗k9k7βAbMIM∗∗μH+πJk6,RA∗∗=αγAAk9IAJ∗∗k7βAbMIM∗∗μH+γAAk9IAJ∗∗k7πJk6+γAAk9εAσAEJ∗∗βAbMIM∗∗μH+γAAk9εAσAEJ∗∗πJk6+γAAk9εAσAβAbMIM∗∗μHSJ∗∗+γSAISJ∗∗k7βAbMIM∗∗μHk8+γSAISJ∗∗k7πJk6k8+γSA1−εJσAEJ∗∗βAbMIM∗∗μHk8+γSA1−εJσAEJ∗∗πJk6k8+γSA1−εJσAβAbMIM∗∗μHSJ∗∗k8+RJ∗∗k9k7βAbMIM∗∗μHk8+RJ∗∗k9k7πJk6k8·k10k9k7k8βAbMIM∗∗μH+πJk6−1,SS∗∗=ξπJSA∗∗βSbMIM∗∗μH+πJμS,ES∗∗=ξβSbMIM∗∗μHSA∗∗+βSbMIM∗∗μH+πJμSEA∗∗k11βSbMIM∗∗μH+πJμS,IAS∗∗=ξεSσSβSbMIM∗∗μSSA∗∗+βSbMIM∗∗μH+πJμSεSσSEA∗∗+IAA∗∗k11k12k11βSbMIM∗∗μH+πJμS,ISS∗∗=ξ1−εSσSβSbMIM∗∗μHSA∗∗+βSbMIM∗∗μH+πJμS1−εSσSEA∗∗+ISA∗∗k11k13k11βSbMIM∗∗μH+πJμS,RS∗∗=ξγAS1−εSσSβSbMIM∗∗μHSA∗∗+γASβSbMIM∗∗μH+πJμS1−εSσSEA∗∗+ISA∗∗k11+γSS1−εSσSβSbMIM∗∗μHSA∗∗+γSSβSbMIM∗∗μH+πJμS1−εSσSEA∗∗+ISA∗∗k11+RA∗∗k11k13βSbMIM∗∗μH+πJμSμSk13k11βSbMIM∗∗μH+πJμS−1,SM∗∗=πMλM∗∗+μM,EM∗∗=λM∗∗πMk14λM∗∗+μM,IM∗∗=σMλM∗∗πMk14μMλM∗∗+μM.

### G. Proof of Theorem 7

Proof The proof is based on using the centre manifold theory [51], as described in [58]. It is convenient to make the following simplification and change of variables. Let *S*_*J*_ = *x*_1_, *E*_*J*_ = *x*_2_, *I*_*AJ*_ = *x*_3_, *I*_*SJ*_ = *x*_4_, *R*_*J*_ = *x*_5_, *S*_*A*_ = *x*_6_, *E*_*A*_ = *x*_7_, *I*_*AA*_ = *x*_8_, *I*_*SA*_ = *x*_9_, *R*_*A*_ = *x*_10_, *S*_*S*_ = *x*_11_, *E*_*S*_ = *x*_12_, *I*_*AS*_ = *x*_13_, *I*_*SS*_ = *x*_14_, *R*_*S*_ = *x*_15_, *S*_*M*_ = *x*_16_, *E*_*M*_ = *x*_17_, and *I*_*M*_ = *x*_18_ so that *N*_*X*_ = *x*_1_ + *x*_2_ + *x*_3_ + *x*_4_ + *x*_5_ + *x*_6_ + *x*_7_ + *x*_8_ + *x*_9_ + *x*_10_ + *x*_11_ + *x*_12_ + *x*_13_ + *x*_14_ + *x*_15_. Using the vector notation **x** = (*x*_1_, *x*_2_, *x*_3_, *x*_4_, *x*_5_, *x*_6_, *x*_7_, *x*_8_, *x*_9_, *x*_10_, *x*_11_, *x*_12_, *x*_13_, *x*_14_, *x*_15_, *x*_16_, *x*_17_, *x*_18_)^*T*^, model (29) can be written in the form *d* **x**/*dt* = **m**(**x**), where **m** = (*m*_1_, *m*_2_, *m*_3_, *m*_4_, *m*_5_, *m*_6_, *m*_7_, *m*_8_, *m*_9_, *m*_10_, *m*_11_, *m*_12_, *m*_13_, *m*_14_, *m*_15_, *m*_16_, *m*_17_, *m*_18_)^*T*^, as follows:

(G.1)
dx1dt=m1=πJ−βJbMx18x1NX−k1x1,dx2dt=m2=βJbMx18x1NX−k2x2,dx3dt=m3=εJσJx2−k3x4,dx4dt=m4=1−εJσJx2−k4x3,dx5dt=m5=γAJx4+γSJx3−k5x5,dx6dt=m6=βAbMx18x6NX−k6x6,dx7dt=m7=βAbMx18x6NX−k7x7,dx8dt=m8=αx3+εAσAx7−k8x8,dx9dt=m9=αx4+1−εAσAx7−k9x9,dx10dt=m10=αx5+γAAx8+γSAx9−k10x10,dx11dt=m11=ξx6−βSbMx18x11NX−μHx11,dx12dt=m12=ξx7+βSbMx18x11NX−k11x12,dx13dt=m13=ξx8+εSσSx12−k12x13,dx14dt=m14=ξx9+1−εSσSx12−k13x14,dx15dt=m15=ξx10+γASx14+γSSx14−μSx15,dx16dt=m16=πM−βMbMx3+x4+x8+x9+x13+x14x16NX−μMx16,dx17dt=m17=βMbMx3+x4+x8+x9+x13+x14x16NX−k14x17,dx18dt=m18=σMx17−μMx18.

The Jacobian of the transformed system (G.1) at the disease-free equilibrium *ℰ*_1_, is given by

(G.2)
$$\begin{array}{c} J\left(\;E_{1}\;\right)=\left(\;J_{1}\;|\;J_{2}\;\right), \end{array}$$

where

(G.3)

Consider the case when *ℛ*_0_ = 1. Suppose, further, that *β*_*M*_ is chosen as a bifurcation parameter. Solving (29) for *β*_*M*_ from *ℛ*_0_ = 1 gives *β*_*M*_ = *β*_*M*_^*∗*^. The transformed system (G.1) at the DFE evaluated at *β*_*M*_ = *β*_*M*_^*∗*^ has a simple zero eigenvalue (and all other eigenvalues having negative real parts). Hence, the centre manifold theory [51] can be used to analyze the dynamics of (G.1) near *β*_*M*_ = *β*_*M*_^*∗*^. In particular, the theorem in [58] (see also [42, 51, 52]) is used. To apply the theorem, the following computations are necessary (it should be noted that we are using *β*_*M*_ instead of *ϕ* for the bifurcation parameter).
*Eigenvectors of J*(*ℰ*_1_)|_*β*_*M*_=*β*_*M*_^*∗*^_. The Jacobian of (G.1) at *β*_*M*_ = *β*_*M*_^*∗*^, denoted by *J*(*ℰ*_1_)|_*β*_*M*_=*β*_*M*_^*∗*^_, has a right eigenvector (associated with the zero eigenvalue) given by

(G.4)
w=w1,w2,w3,w4,w5,w6,w7,w8,w9,w10,w11,w12,w13,w14,w15,w16,w17,w18T,

where

(G.5)
w1=−βJbMw18x1k1x1+x6+x11,w2=βJbMw18x1k2x1+x6+x11,w3=εJσJw2k3,w4=1−εJσJw2k4,w5=γAJw3+γSJw4k5,w6=1k6αw1−bMβAw18x6x1+x6+x11,w7=1k7αw2+bMβAw18x6x1+x6+x11,w8=αw3+εAσAw7k8,w9=αw4+1−εAσAw7k9,w10=αw5+γAAw8+γSAw9k10,w11=1μSξw6−bMβSw18x11x1+x6+x11,w12=1k11ξw7+bMβSw18x11x1+x6+x11,w13=ξw8+εSσSw12k12,w14=ξw9+1−εSσSw12k13,w15=ξw10+γAA+γSAw14μS,w16=−bMβMx16μMw3+w4+w8+w9+w13+w14x1+x6+x11,w17=x11bMβMk9w3+w4+w8+w9+w13+w14x1+x6+x11,w18=σMμM.

Also, *J*(*ℰ*_1_)|_*β*_*M*_=*β*_*M*_^*∗*^_ has a left eigenvector **v** = (*v*_1_, *v*_2_, *v*_3_, *v*_4_, *v*_5_, *v*_6_, *v*_7_, *v*_8_, *v*_9_, *v*_10_, *v*_11_, *v*_12_, *v*_13_, *v*_14_, *v*_15_, *v*_16_, *v*_17_, *v*_18_) (associated with the zero eigenvalue), where

(G.6)

*Computations of Bifurcation Coefficients a and b*. The application of the theorem in [58] entails the computation of two bifurcation coefficients *a* and *b*. It can be shown, after some algebraic manipulations, that

(G.7)

Furthermore,

(G.8)
bv17∑i=118wi∂2f17∂xi∂βp∗=v17x16bMw3+w8+w9+w13+w14x1+x6+x11>0.

Hence, it follows from Theorem 4.1 of [58] that the transformed model (G.1) (or, equivalently, (10)) undergoes backward bifurcation at *ℛ*_0_ = 1 whenever the following inequality holds:

(G.9)
$$\begin{array}{c} a>0. \end{array}$$
